# Lipidomic insights into the reaction of baking lipases in cakes

**DOI:** 10.3389/fnut.2023.1290502

**Published:** 2023-12-12

**Authors:** Charlotte Dorothea Stemler, Sabrina Geisslitz, Adele Cutignano, Katharina Anne Scherf

**Affiliations:** ^1^Department of Bioactive and Functional Food Chemistry, Institute of Applied Biosciences, Karlsruhe Institute of Technology (KIT), Karlsruhe, Germany; ^2^Istituto di Chimica Biomolecolare (ICB), Consiglio Nazionale delle Ricerche (CNR), Pozzuoli (Napoli), Italy

**Keywords:** baking, glyceroglycolipid, lipids, substrate specificity, UHPLC-MS

## Abstract

Lipases are promising improvers of cake batter and baking properties. Their suitability for use in various cake formulations cannot be predicted yet, because the reactions that lead to macroscopic effects need to be unravelled. Therefore, the lipidome of three different cake recipes with and without lipase treatment was assessed by ultra high performance liquid chromatography-mass spectrometry before and after baking. By comparing the reaction patterns of seven different lipases in the recipes with known effects on texture, we show that lipase substrate specificity impacts baking quality. Key reactions for the recipes were identified with the help of principal component analysis. In the eggless basic cake, glyceroglycolipids are causal for baking improvement. In pound cake, lysoglycerophospholipids were linked to textural effects. Lipase substrate specificity was shown to be dependent on the recipe. Further research is needed to understand how recipes can be adjusted to achieve optimal lipase substrate specificity for desirable batter and baking properties.

## 1 Introduction

Lipases are beneficial replacements for traditional surfactants in baking, because they lead, e.g., to improved textural characteristics and a prolonged crumb softness. At the same time the use of lipases is clean-label, cheaper and more effective than, e.g., mono- and diacetyl tartaric acid esters of mono- and diglycerides of fatty acids (DATEM), one of the most used surfactants in bakery (1, 2). Bread baking is therefore one of the main fields of application for lipases and especially for phospholipases (3). However, only scarce data is available on the possible applications of lipases in bakery products other than bread.

Recently, the use of lipases as baking improvers for cakes has been explored (2, 4). Seven lipases were applied in three different recipes, namely an eggless basic cake, a traditional pound cake and a yeast-based brioche. In cake dough and batters, single lipases led to a reduction of density, less stickiness and a higher liquefaction of the batter. The extent of improvement to batter quality depended both on the lipase and the recipe. The effects were greatest in basic cake, less pronounced in pound cake and nearly no improvements occurred in brioche (4). Similar trends were observed in the baked products, where especially the texture in terms of firmness, springiness, resilience and cohesiveness was affected. In basic cake, three out of seven lipases lead to softer products while also reducing the cohesiveness and resilience. In pound cake, the extent of texture improvement was less than in basic cake. Still, several lipases reduced the firmness and cohesiveness of the cakes. In brioche, only resilience and cohesiveness were affected to small extents (2). The differences between the lipases were greater than known for lipase effects in bread (2, 4, 5). This may be due to the different ingredients used in bread and cakes. Compared to bread, the range of available substrates for lipase reactions is more complex. Besides the substrates, also the type of lipase used might play a role in its macroscopic effect.

Lipases can be subdivided into several groups according to their preferred substrates, namely triacylglycerol (TG)-lipases (EC 3.1.1.3), galactolipases (EC 3.1.1.26) and different phospholipases (e.g., EC 3.1.1.4, EC 3.1.1.5, EC 3.1.1.32). However, most lipases show broad specificities and act on a wide range of substrates (1). To achieve the desired effects like an increase in loaf volume in bread, lipases need to catalyze the hydrolysis of glyceroglycolipids and glycerophospholipids in a way that both polar reaction products and either unmodified lipids or non-polar lipids act in synergistic ways (6). To capture these reaction products, new analytical methods are needed.

Lipidomics has evolved as a powerful tool to capture the complete set of lipids (the lipidome) and their interactions with other molecules in a given cell or organism. Lipidomic techniques are also applied more and more for the analysis of food, e.g., for research on dietary lipids and nutrition or for food authenticity and food safety (7). The insights on the composition and structure of lipids within complex food matrices can be used to characterize lipase reactions, as recently done by Xuan et al. (8). They applied mass spectrometry to analyze the lipid modification by lipases in oil. Especially in light of the influence of the matrix on lipase substrate specificity for fatty acids of different chain lengths as shown recently by our group (9), a deeper knowledge about lipase reactions in food is of great interest. Target species for lipidomic research in cake originate from wheat, eggs and/or milk fat.

**Figure 1:**
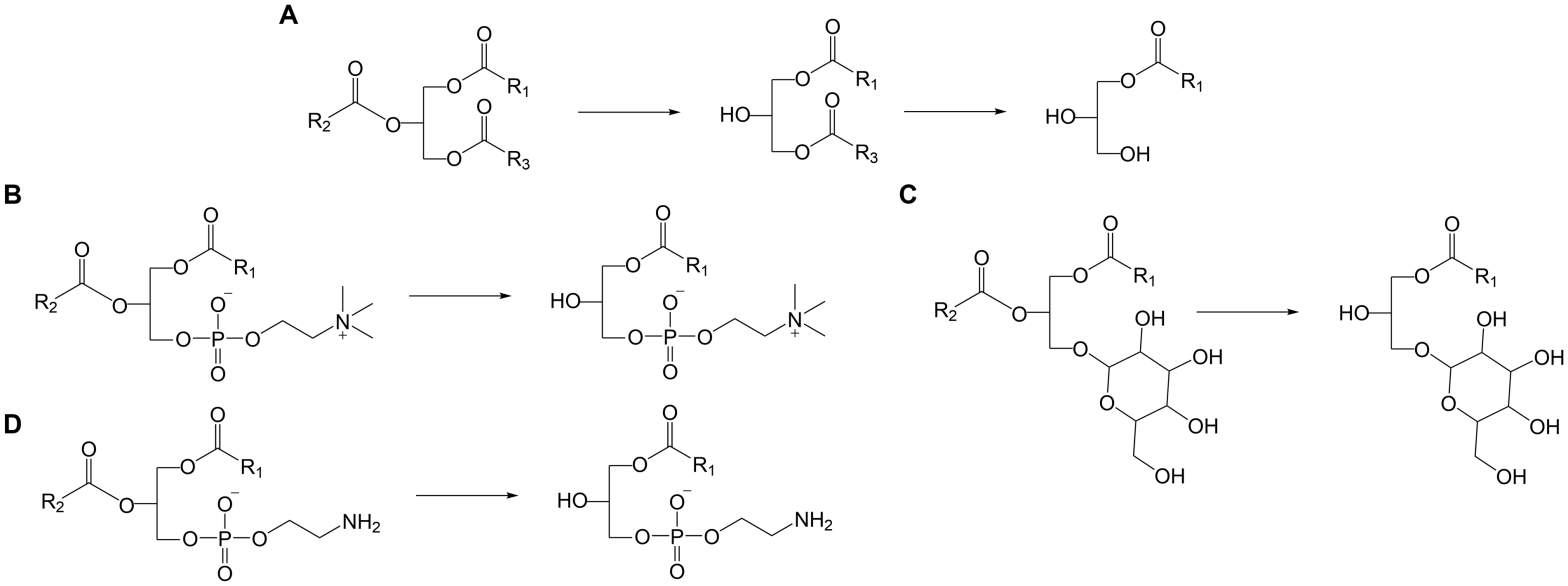
Examples of possible lipase reaction mechanisms. **(A)** Hydrolysis of TG to DG and MG, **(B)** hydrolysis of PC to LPC, **(C)** hydrolysis of PE to LPE **(D)** hydrolysis of MGDG to MGMG.

Lipids originating from wheat flour comprise neutral lipids such as fatty acyls (FA) and glycerolipids as well as polar lipids like glycerophospholipids and glyceroglycolipids (10). Lipidomic studies on wheat flour lipids reported up to 85 different lipid species (11), including FA, the glycerolipids diacylglycerols (DG, Figure 1) and TG (Figure 1), the glyceroglycolipids digalactosyldiacylglycerol (DGDG) and monogalactosyldiacylglycerol (MGDG, Figure 1) and the glycerophospholipids glycerophosphocholine (PC, Figure 1), glycerophosphoethanolamine (PE, Figure 1), glycerophosphoinositol (PI), glycerophosphoglycerol (PG), glycerophosphoserine (PS), glycerophosphate (PA), and lysoglycerophosphocholine (LPC, Figure 1) (10–12). Additionally, *N*-acyl glycerophosphoethanolamine (NAPE) and *N*-acyl lysoglycerophosphoethanolamine (NALPE) were identified in wheat flour, bread dough and bread crumb (13).

In addition, cake contains eggs and/or milk fat from butter as major sources of lipids. The composition of milk fat is dominated by TG (98% of total lipids) (14). In addition, it is composed of DG, monoacylglycerols (MG), cholesterol, FA, PC, PE, PG, PS, PI, PA, LPC, lysoglycerophosphoethanolamine (LPE), ceramides, sphingomyelins, and glycosylceramides (14–17). Up to 45 different fatty acids have been reported for milk fat with chain lengths between C2:0 – C22:0 (18, 19). Eggs can be divided in egg white and egg yolk. Egg white contains only about 0.02% lipids while they make up approximately 30% of egg yolk (20, 21). Research on egg lipids therefore usually focuses on the lipids from egg yolk. They comprise mostly TG (66%) but also include about 33% of glycerophospholipids (PC, PE, PG, PI, PS, LPC, and LPE) in addition to cholesterol and sphingomyelin (21–24). Both butter and eggs therefore enlarge the pool of possible substrates compared to wheat flour alone, resulting in a large variety of reaction products when lipases are added. An overview of all main lipid classes reported for the ingredients of cake, the lipid category they belong to and their respective hydrolysis reaction products is given in Supplementary Figure S1 (10–16, 21, 24).

The relationship between different lipid classes and the resulting impact on cake batter and baking quality remains unknown. We recently showed that differences in lipase substrate specificity are most likely the decisive factor for differences in lipase effectiveness (2, 4). Our hypothesis is that by analyzing the substrate specificity of lipases we can establish a connection between specific lipase reactions in cakes and their macroscopic effects such as liquefaction of cake batter and inhibition of staling. For this we rely on previous research on the impact of lipases on both cake batter and cake baking quality in cakes of three different recipes (2, 4). The recipes included an eggless basic cake, a traditional pound cake and a yeast-based brioche. The aim of our work was to analyze the lipid composition using ultra high performance liquid chromatography-mass spectrometry (UHPLC-MS) in both cake batter and the final products of the three recipes as affected by lipase addition. Thereby a better understanding of lipase reactions and their consequences in cakes and the identification of optimal lipase substrate specificity patterns are to be achieved.

## 2 Materials and methods

### 2.1 Reagents and ingredients

All chemicals and reagents were of analytical grade or higher. The lipid standard 1,2,3-triheptadecanoyl-*sn*-glycerol (TG 17:0/17:0/17:0) was from Merck KGaA (Darmstadt, Germany). All ingredients for batter/dough making and baking were of commercial quality and either bought at a local supermarket (pasteurized whole eggs, baking powder, yeast, and butter) or kindly donated by Dr. August Oetker Nahrungsmittel KG (Bielefeld, Germany), including wheat flour type 405 (Good Mills GmbH, Hamburg, Germany) and extra-white powdered sugar (Nordzucker, Braunschweig, Germany). To maintain the original lipid composition, butter and eggs were frozen directly after purchase and stored at −20°C.

### 2.2 Lipases

Lipases were chosen according to previous research (2, 4). In brief, seven lipases already characterized in terms of their substrate specificities towards *p*-nitrophenyl esters and emulsified butter as well as their activities in commercially available lipase activity kits were used (9). Their effects on the properties of cake batters and products for three different recipes were known from previous experiments (2, 4). They were named A, E, G, J, K, M, and O in line with previous publications. Lipases A, E, J, and M were declared as phospholipases by the manufacturers, lipase K as glycolipase and lipase O as TG-lipase. The type of lipase was unknown for lipase G. Lipase addition was performed as described in Stemler and Scherf (2). For basic cake and pound cake this included batter-based dosages of 400 mg/kg (lipase A), 500 mg/kg (lipase E), 300 mg/kg (lipases G and K), 70 mg/kg (lipase J) and 200 mg/kg (lipases M and O). For brioche, 30% of those dosages were applied on a flour base. To reduce weighing errors, lipases were dissolved in water prior to addition and added by volume. For the control samples, the same volume of water was added.

### 2.3 Sample preparation

Cake batter preparation and cake baking were performed according to Stemler and Scherf (2). All samples were prepared in triplicate. Basic cake, pound cake and brioche were manufactured using a commercial food processor with planetary mixing (Robert Bosch GmbH, Stuttgart, Germany) equipped with a whisk and a kneading hook.

Basic cake batter preparation consisted of two steps: first creaming 100 g of butter, 50 g of sugar and 2.5 g of salt and then the addition and mixing in of 200 mL of water, 250 g of wheat flour and 15 g of baking powder. Afterwards, the lipases were added as indicated above.

For pound cake, equal amounts (200 g) of butter and sugar were mixed with 2 g of salt. Then, 200 g of eggs and a mixture of 200 g of wheat flour and 0.6 g of baking powder were added and blended in successively. Afterwards, the lipases were added as indicated above. Incubation with lipases for basic cake and pound cake was for 1 h at 22°C.

For brioche, a pre-dough including 300 g of wheat flour, 125 mL of lukewarm water, 35 g of fresh yeast and 50 g of pasteurized egg was incubated for 2 h with lipases in a proofing cabinet (37°C). Then, another 100 g of butter, 40 g of sugar, 160 g of flour and 4 g of salt were kneaded in and the samples were given another 20 min of proofing time before either freezing (dough samples) or baking (cake samples).

The lipase-treated and control batters were frozen directly after incubation with lipases. For cake samples, muffins of 50 g batter weight each were baked in a preheated hot air oven (UNOX Deutschland GmbH, Büren, Germany) at 180°C for 12 min and left to cool for 2 h at 22°C. All samples were lyophilized, milled and stored at −20°C until further analysis. A schematic overview of all samples can be found in Supplementary Figure S2.

### 2.4 Lipid extraction

Lipid extraction was performed according to Cutignano et al. (25) based on a procedure first established by Matyash et al. (26). The sample (1 mg) was thoroughly mixed with 300 μL of methanol. Then, 1 mL of 2-methoxy-2-methylpropane (methyl *tert-*butyl ether, MTBE) was added, the mixture was sonicated for 5 min and then shaken gently at 22°C for 1 h. Phase separation was induced by the addition of 250 μL of deionized water and shaking for 10 min. After centrifugation (10 min, 4°C, 3,000 *g*) the upper organic phase was transferred into a pre-weighed glass vial. The lower phase including the pellet was re-extracted with 300 μL MTBE after sonication by gentle shaking at 22°C for 30 min. The mixture was then centrifuged and the organic phases were pooled before drying under nitrogen. Dried extracts were stored at −20°C until further analysis.

### 2.5 Lipid analysis

Lipids were redissolved in 3 mL of a mixture of methanol and 2-propanol (1,1, v:v) and analyzed via UHPLC-MS according to Cutignano et al. (27). The operating conditions are given in Supplementary Table S1. The procedure was established for basic cake samples on a Q Exactive Hybrid Quadrupole-Orbitrap (Thermo Fisher Scientific, Waltham; United States) and then transferred to a Q-Exactive Plus Orbitrap (Thermo Fisher Scientific) for pound cake and brioche. The comparability of both systems was assured by comparing the results for chosen samples of all recipes and in detail for the quality control samples of basic cake. Quality control samples were prepared batch-wise (all batter samples of one recipe or all cake samples of one recipe) and consisted of equal amounts of all 24 samples from the respective batch (see also Supplementary Figure S2).

For quantification, the most abundant lipid class (TG) was chosen as reference and TG 17:0/17:0/17:0 was added for semiquantitative analysis as internal standard prior to extraction to a final concentration of 3 μg/mL. For the determination of recovery, for each triplicate of samples a fourth sample was extracted in which the internal standard was added after extraction before analysis. The calculated recoveries are listed in Supplementary Table S2.

### 2.6 Lipid identification and statistical analysis

For lipid identification and quantification, the software LipidSearch (version 5.0.63.7, Thermo Fisher Scientific) was used. The samples of each batch were aligned. The quality control samples were included in the alignments to improve lipid identification. All identified lipids were manually double-checked for reliability of the identification and integration area.

For the calculation of substrate specificity ratios, the peak area of each individual lipid was normalized by the respective sample weight and the recovery of the sample using Microsoft Excel (Microsoft Office Professional Plus 2019, Microsoft Corporation, Redmond, WA, United States). The recovery of the sample was determined as the recovery of the internal standard and calculated as the ratio of the peak area of the internal standard in the sample spiked prior to extraction to its area in the corresponding sample spiked after extraction.


$$\mathrm{normalized}\;\mathrm{peak}\;\mathrm{area}=\frac{\mathrm{peak}\;\mathrm{area}\;}{\;\mathrm{sample}\;\mathrm{weight}\;\cdot \;\mathrm{recovery}\;}$$

Then the normalized peak area of each lipid calculated as above in lipase-treated samples was divided by the corresponding normalized peak area of the same lipid in the corresponding control sample. The average value of the three replicates (three different batters of the same recipe) was then referred to as “lipase specificity lipid ratio,” short “ratio” of each lipase in the respective matrix, e.g., the ratio of lipase A for TG 12:0_14:0_16:0 in basic cake batter.


$$\mathrm{ratio}=\frac{\mathrm{normalized}\;\mathrm{peak}\;\mathrm{area}\;\mathrm{in}\;\mathrm{lipase}-\mathrm{treated}\;\mathrm{sample}}{\;\mathrm{normalized}\;\mathrm{peak}\;\mathrm{area}\;\mathrm{in}\;\mathrm{control}\;\mathrm{sample}}$$

For intact lipids, as, e.g., TG, a ratio below 1 implies the preferred hydrolysis of the lipid and a ratio greater than 1 stands for a lack of hydrolysis by the lipase. For lysolipids, a ratio greater than 1 indicates a release and thereby formation of the lysolipid by lipases. The ratio of intact lipids is inversely proportional to the specificity, i.e., low ratios correspond to high specificities while high ratios to a lack of specificity.

All ratios were combined batch-wise in a new matrix using Microsoft Excel and processed for principal component analysis (PCA) with Origin Pro 2022b (OriginLab Corporation, Northampton, MA, United States).

## 3 Results

A total of 22 lipid classes were detected in the samples (Supplementary Figure S1 and Supplementary Table S3). The experiments showed that the chosen conditions were well suitable to analyze all the main lipid classes including PC, PE, PS, LPC, LPE, DG, TG, and sphingomyelin. A complete list of identified lipid classes and the corresponding polarity settings as well as the chosen adducts for quantification can be found in Supplementary Table S3.

### 3.1 Basic cake

#### 3.1.1 Batter

In basic cake batter, 105 different lipid species were identified (Supplementary Table S4). The highest number of lipid species (81.0%) was attributable to TG. Besides TG, the samples contained DG (7.6%), LPC (1.9%), PE (1.0%) and sphingosine (4.8%). In addition, several glyceroglycolipids were found, namely DGDG 16:0_18:2, DGDG 18:2_18:2, digalactosylmonoacylglycerol (DGMG) 18:2 and monogalactosylmonoacylglycerol (MGMG) 18:2. The fatty acids of all lipid classes within the batter samples ranged from 4:0 to 26:0.

**Figure 2:**
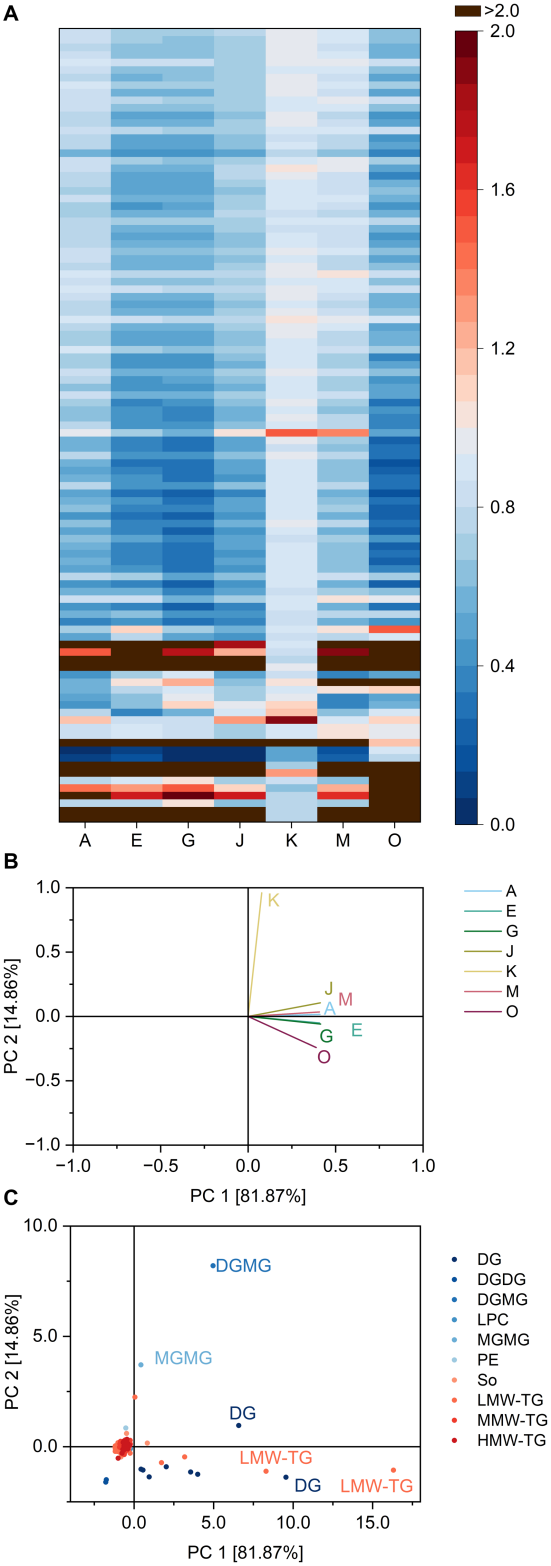
Substrate specificity of the lipases A, E, G, J, K, M and O towards different lipids in basic cake batter. For a complete list of substrates, refer to Supplementary Table S4. The order of lipids therein corresponds to the one in the heatmap. **(A)** Lipase specificity ratios depicted as heatmap, **(B)** Loading plot of the principal component analysis (PCA), **(C)** Scores plot of the PCA. Data is shown as mean of three replicates and for abbreviations please refer to the text.

The ratios are depicted as a heatmap for all combinations of lipids and lipases in Figure 2 and show possible similarities and differences between the lipase reaction patterns. The ratios averaged over all DG ranged from 0.8 (lipase K) to 6.2 (lipase O), indicating a preferred formation of DG by lipase O. Lipase K either hydrolyzed both TG and DG with similar ratios or did not interact with either of them. This question can be solved by analyzing the ratios of lipase K for TG. To better process the large amount of TG and to take into consideration possible differences in fatty acid specificity of the lipases, the TG were further divided into three groups according to the number of carbon atoms within fatty acid residues. The groups comprise low molecular weight TG (LMW-TG) with up to 40 carbon atoms, medium molecular weight TG (MMW-TG) with 40–54 carbon atoms and high molecular weight TG (HMW-TG) for all TG with more than 54 carbon atoms. Lipase K showed only weak interactions with all three groups, leading to average ratios of 0.9 and probably low specificity towards TG in general. Compared to the other lipases, the impact of lipase K on TG was smaller. For MMW-TG and HMW-TG, the average ratios of the remaining lipases varied from 0.6 (lipases E, G and O) to 0.8–0.9 (lipase M). Within LMW-TG, the share of the four lightest molecules identified (TG 4:0_8:0_10:0, TG 6:0_8:0_10:1, TG 4:0_10:0_10:0 and TG 4:0_8:0_14:1) was increased by all lipases (leading to ratios from 1.2 to 15.1), except by lipase K. The remaining LMW-TG were hydrolyzed preferentially by all other lipases (average ratios 0.4–0.7). This indicates that, if a lipase interacts with TG in basic cake batter, it mostly leads to the hydrolysis of TG with 26–40 carbon atoms within the fatty acid residues. Lipase K interacted only weakly with TG and therefore also with DG.

The different behavior of lipase K compared to the other lipases was highlighted both in the heatmap and the loading plot of the PCA (Figure 2). The two principal components accounted for 96.7% of total variance between the lipases and clearly discriminated lipase K from the other lipases. Besides LMW-TG 4:0_8:0_10:0 and 6:0_8:0_10:1 and DG 18:1_12:0 and 18:1_18:2, this was also due to its release of the glyceroglycolipids DGMG 18:2 and MGMG 18:2.

#### 3.1.2 Cake

In baked basic cake, a total of 143 different lipid species were identified (Supplementary Table S5). According to expectations based on the lipid distribution in batter, 82.5% of the lipid species were represented by TG. Compared to the batter, more lipid classes including PE, MG, lysodimethylphosphatidylethanolamine (LdMePE), LPC, LPE and lysoglycerophosphoglycerol (LPG) were detected. However, DG were not present in the extracts.

**Figure 3:**
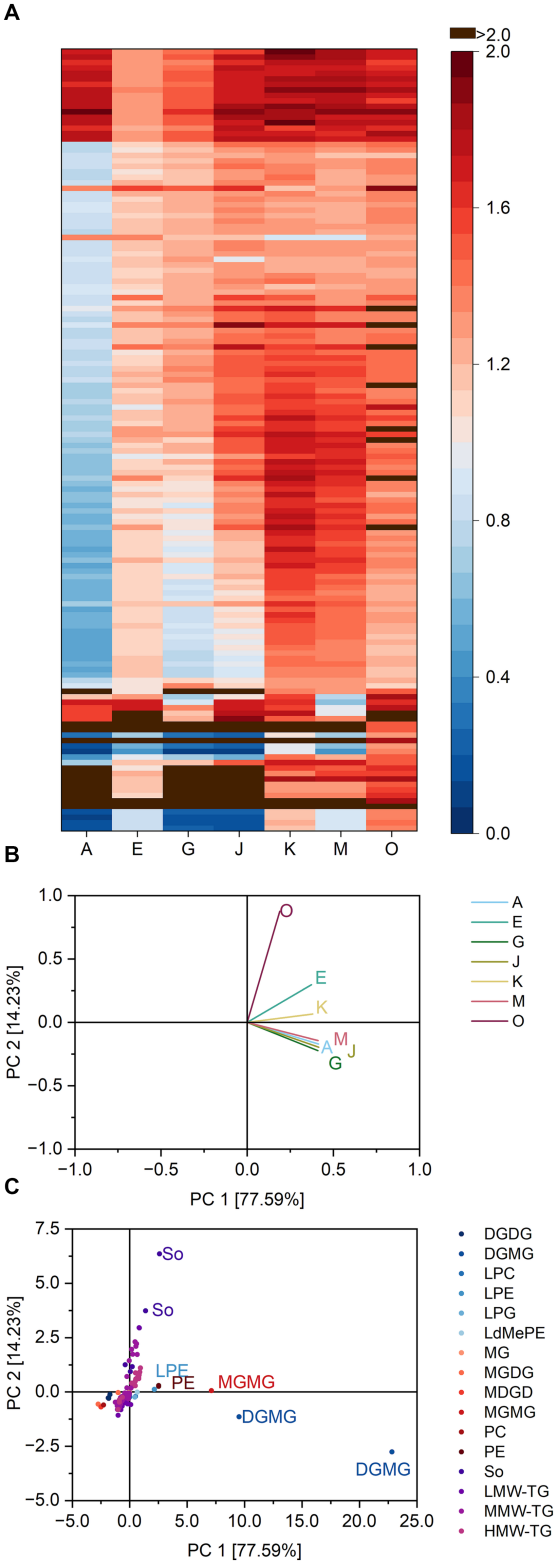
Substrate specificity of the lipases A, E, G, J, K, M and O towards different lipids in basic cake samples. For a complete list of substrates, refer to Supplementary Table S5. The order of lipids therein corresponds to the one in the heatmap. **(A)** Lipase specificity ratios depicted as heatmap, **(B)** Loading plot of the principal component analysis (PCA), **(C)** Scores plot of the PCA. Data is shown as mean of three replicates and for abbreviations please refer to the text.

Again, the lipases varied in their specificity ratios (Figure 3 and Supplementary Table S5). Especially their reactivity towards DGDG resulting in the formation of DGMG differed greatly. Although the share of DGMG was increased by all lipases, the corresponding ratios varied between 1.8 (lipase O towards DGMG (18:2)) and 39.2 (lipase G towards DGMG 18:3). The lipases A, G and J were most active towards DGMG and all had ratios greater than 13.2. They also led to higher shares of lysoglycerophospholipids (average ratios to LPC 16:0, LPC 18:3, LPC 18:2, LPE 16:0, LPG 16:0 and LdMePE 18:2 were 3.0, 4.5, and 3.4 for A, G, and J, respectively) compared to the other lipases (average ratios of 1.2, 1.5, 1.5, and 1.6 for E, K, M, and O, respectively).

The PCA (variance 91.8%) clustered the lipases A, G, M, and J (Figure 3). Their similarities were mostly due to their specificity towards DGMG 18:2 and 18:3. The reactivity towards ceramides resulting in the release of sphingosine (So d12:0;pO) was statistically the most important unique feature of lipase O.

### 3.2 Pound cake

#### 3.2.1 Batter

Due to the additional presence of eggs in the recipe, pound cake batter contained a broader range of lipids, resulting in the detection of 249 different species (Supplementary Table S6). Besides 60.2% of TG species, several glyceroglycolipids, sphingolipids, glycerophospholipids and their corresponding lyso-forms were identified. The glycerophospholipids PA, PC, PE, and glycerophosphoethanol (PEt) made up 20.1% of all species; the lysoglycerophospholipids LPC and LPE another 8.0%.

**Figure 4:**
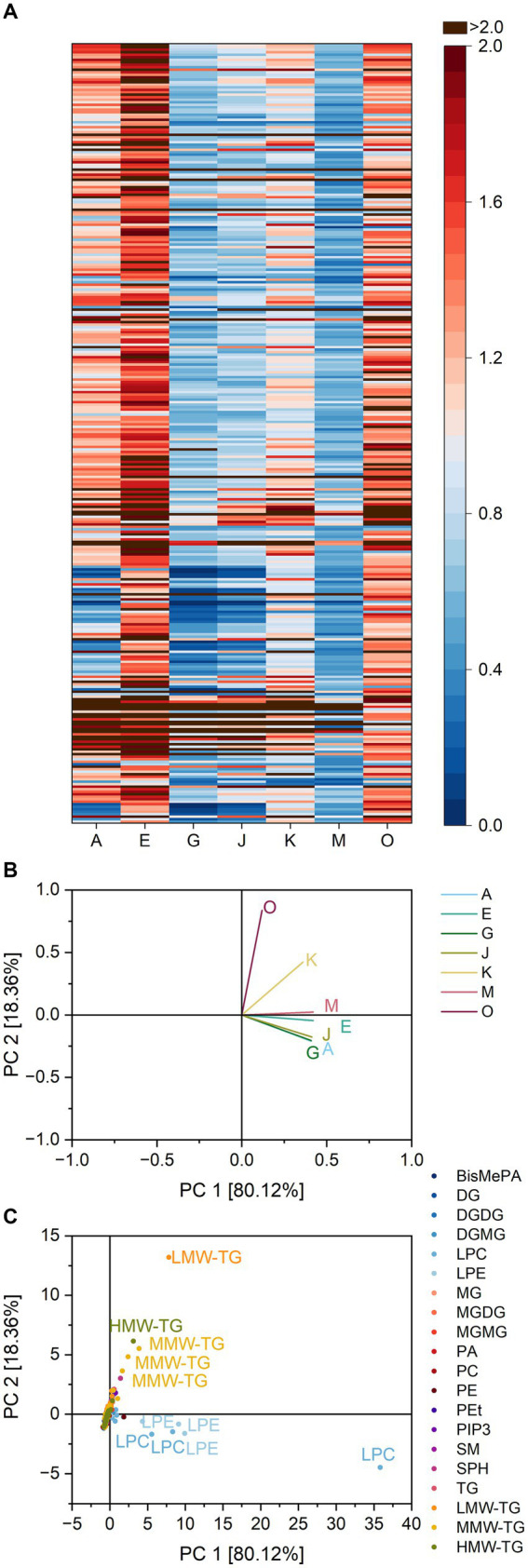
Substrate specificity of the lipases A, E, G, J, K, M and O towards different lipids in pound cake batter. For a complete list of substrates, refer to Supplementary Table S6. The order of lipids therein corresponds to the one in the heatmap. **(A)** Lipase specificity ratios depicted as heatmap, **(B)** Loading plot of the principal component analysis (PCA), **(C)** Scores plot of the PCA. Data is shown as mean of three replicates and for abbreviations please refer to the text.

The heatmap of all lipase specificity ratios within pound cake batter is depicted in Figure 4. The lipases differed in their ratios towards different lipid classes. Concerning PC, for example, the lipases A, G and J were most active and had ratios as low as 0.11 (lipase A towards PC 16:0_18:2), 0.14 (lipase G towards PC 16:0_20:4) and 0.07 (lipase J towards PC 16:0_18:2). Lipase J had a high impact on the share of lysoglycerophospholipids in general with an average ratio of 12.3 compared to, e.g., 6.3 for lipase K and 0.7 for lipase O. Lipase J also led to the formation of DGMG and MGMG (ratios of 3.6 and 1.9) and correspondingly to the loss of DGDG and MGDG (0.1 and 0.0).

The PCA captured 97.5% of the overall variance in the first two components. Lipase O was clearly discriminated from the other lipases. The lipases A, G, and J were clustered in the fourth quadrant, while the reaction patterns of the lipases E, M and K led to their clustering in the second quadrant close to J, G, and A. Similarities of J, G, and A were mostly due to the release of LPC 18:1, 22:6, 22:5 and 20:4 and LPE 18:1, 22:5 and 20:5, while the other lipases were characterized by their influence on the share of LMW-TG 4:0_6:0_12:0 and 4:0_6:0_14:1.

#### 3.2.2 Cake

The number of extractable lipids from baked pound cake was 316 (Supplementary Table S7). Again, about two thirds of all lipid species belonged to TG (59.5%). The identified lipid classes were the same as for the batter, besides glycerophosphoinositol (3,4,5)-trisphosphate (PIP3), which was only detected in the extracts of the baked products.

Again, lipase effects on specific lipid classes showed a wide range of activities (Supplementary Table S7). This can be shown exemplarily for the three lipases with the highest activity within baked pound cake: While the formation of lysoglycerophospholipids was catalyzed with an average ratio of 24.3 by lipase G, the corresponding value for lipase K was 3.2. For lipase O it was 1.3, indicating a weak tendency for the formation. A smaller range of ratios was found for the formation of DG (0.7 for lipase G, 1.0 for lipase K and 1.3 for lipase O) (Supplementary Table S7).

**Figure 5:**
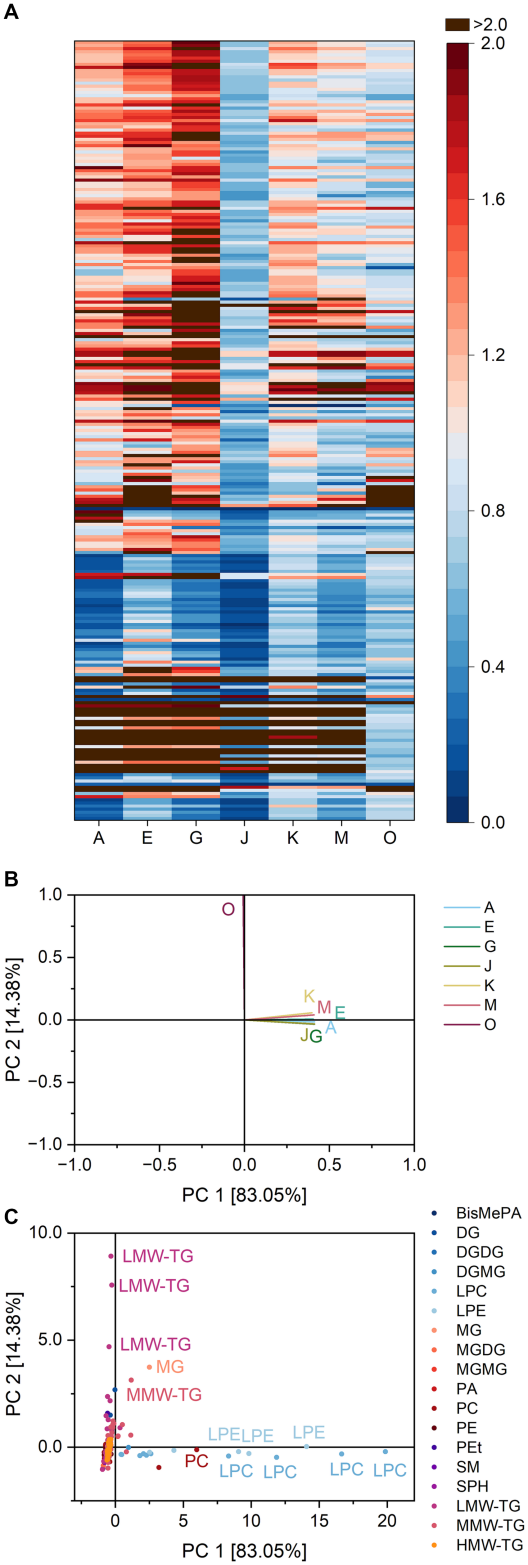
Substrate specificity of the lipases A, E, G, J, K, M and O towards different lipids in pound cake samples. For a complete list of substrates, refer to Supplementary Table S7. The order of lipids therein corresponds to the one in the heatmap. **(A)** Lipase specificity ratios depicted as heatmap, **(B)** Loading plot of the principal component analysis (PCA), **(C)** Scores plot of the PCA. Data is shown as mean of three replicates and for abbreviations please refer to the text.

Visible similarities in the reaction patterns of the lipases A, G, and J (Figure 5) were confirmed by PCA (variance 97.4%). The lipases A, G, and J were clustered closely together, separated from the lipases E and M. The lipases O and K showed only weak similarities to other lipases. The clustering of the lipases A, G, and J was due to their ratio for LPC 22:6 and 22:5 and LPE 20:4 and 22:6 as already stated for pound cake batter. Lipase O was mostly characterized by its interaction with TG 18:0_18:2_18:2, 16:0_18:2_18:2, 6:0_16:0_18:2 and 8:0_10:0_10:0, while lipase K interacted both with glycerophospholipids and TG.

### 3.3 Brioche

#### 3.3.1 Dough

In brioche dough, 202 different lipid species were identified (Supplementary Table S8). As described for basic cake and pound cake, the majority were TG (70.8%). Besides TG, DG and MG, several glycerophospholipids (Bis-methyl glycerophosphate (BisMePA), PC, PE, PEt, glycerophosphoinositol (4,5)-bisphosphate (PIP2), PIP3) and lysoglycerophospholipids (LPC and LPE) as well as the glyceroglycolipids DGDG/DGMG and MGDG/MGMG, the sphingolipids sphingomyelin and sphingoid bases (SPB) were also present in the extracts from brioche dough.

Differences between the samples (control compared to all lipase-treated samples) were low compared to basic cake and pound cake. For instance, the ratios towards DGDG varied from 0.52 to 2.14 (basic cake batter: 0.02 to 0.88 and pound cake batter: 0.11 to 0.82). The averages of the ratios for lipid classes were closer to 1.0, e.g., varying from 0.4 to 1.4 for lysoglycerophospholipids instead of values greater than 30 as seen in pound cake.

**Figure 6:**
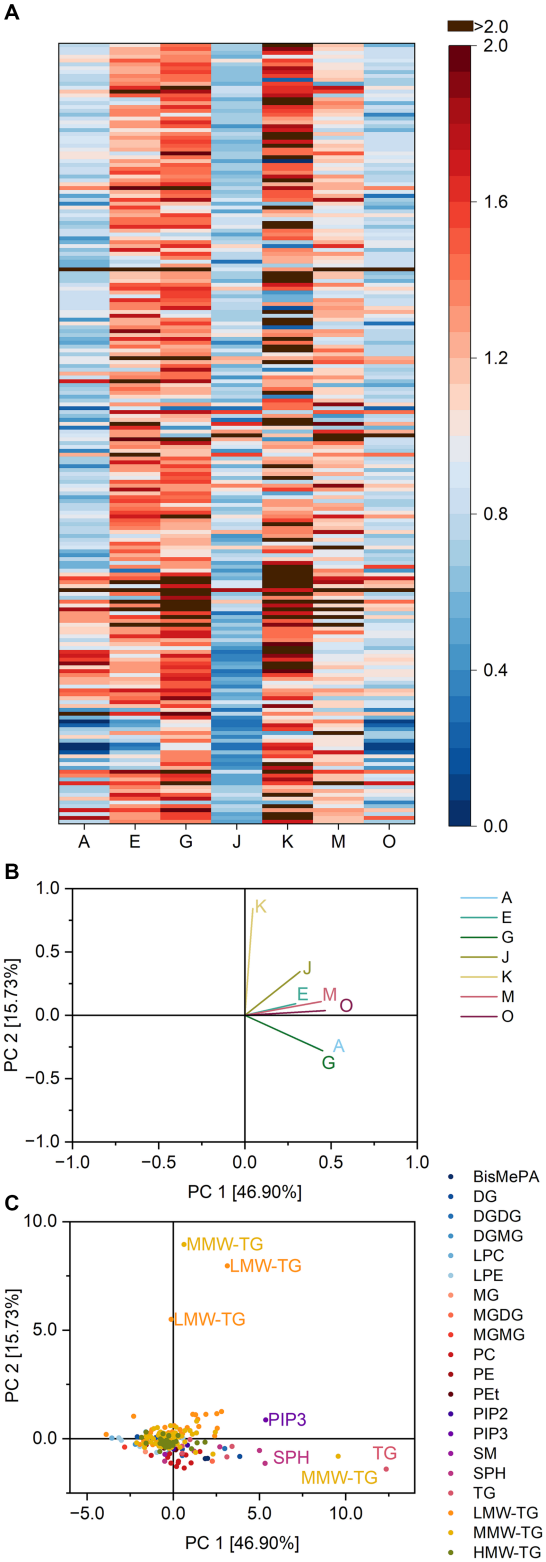
Substrate specificity of the lipases A, E, G, J, K, M and O towards different lipids in brioche dough. For a complete list of substrates, refer to Supplementary Table S8. The order of lipids therein corresponds to the one in the heatmap. **(A)** Lipase specificity ratios depicted as heatmap, **(B)** Loading plot of the principal component analysis (PCA), **(C)** Scores plot of the PCA. Data is shown as mean of three replicates and for abbreviations please refer to the text.

Consequently, the heatmap showing the ratios contains fewer coherent areas, but more individual values varying within lipid classes (Figure 6).

Accordingly, the discrimination of the lipases via PCA included only 62.6% of all variance in the first two components. There seemed to be three clusters (first the lipases A, G; second the lipases J, E, M and O and third lipase K). However, their respective similarities were all due to their ratios for specific TG 10:0_17:1_18:1, 4:0_17:1_18:1, 4:0_14:0_18:2, 14:0_16:0_16:0 and 20:6_21:6_21:6; P and less to SPB d18:0 and PIP3 P-6:0/16:3; P. Compared to basic cake and pound cake there was no clear influence of specific lipid classes such as DGMG for basic cake and LPC/LPE for pound cake.

#### 3.3.2 Cake

In the extracts from baked brioche, 192 different lipid species were present, slightly less than in dough (Supplementary Table S9). However, similar to dough, 70.3% were TG. The remaining lipid classes were also similar, the only difference being the identification of PG in baked brioche which was not present in the extracts from dough.

Differences between the lipases concerning their substrate specificities were again small compared to basic cake and pound cake. The lipases had ratios from 0.5–1.5 towards LPC 18:1 for instance (Supplementary Table S9), which had been released with ratios of up to 13.3 in baked pound cake. This hints at a smaller release of LPC in baked brioche compared to baked pound cake.

**Figure 7:**
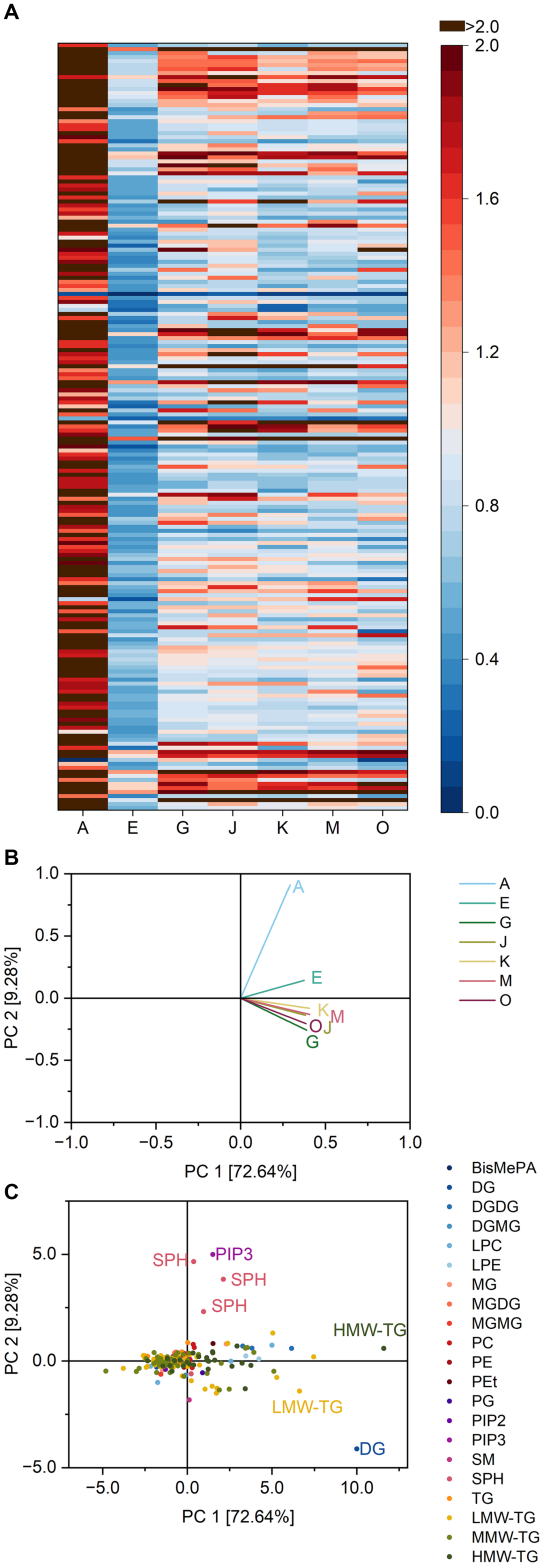
Substrate specificity of the lipases A, E, G, J, K, M and O towards different lipids in brioche samples. For a complete list of substrates, refer to Supplementary Table S9. The order of lipids therein corresponds to the one in the heatmap. **(A)** Lipase specificity ratios depicted as heatmap, **(B)** Loading plot of the principal component analysis (PCA), **(C)** Scores plot of the PCA. Data is shown as mean of three replicates and for abbreviations please refer to the text.

Accordingly, the substrate specificity heatmap of baked brioche lacked clear patterns (Figure 7). Lipase A had visibly overall higher ratios towards most substrates compared to the other lipases.

The PCA for the batch of baked brioche accounted for 81.9% of all variance between the lipases in the two principal components. Lipase A was separated from the other lipases due to its specificity towards SPB d18:0, d14:1 and d16:1. Besides, also DG 42:6; O, TG 4:0_16:0_17:0 and TG 16:0_18:0_24:0 affected the reaction patterns.

## 4 Discussion

### 4.1 Suitability of the method

The method includes the chosen extraction procedure, the measurement settings and data analysis. It was suitable to analyze 22 different lipid classes from lipase-treated cake samples. Especially the dominant class of TG was covered extensively. However, due to the complexity of lipids, it is normally not possible to cover all lipid classes in a single run with one analytical technique.

Several lipid classes have been reported for the individual ingredients of the cakes, but not all of them were identified in the extracts. There are four main reasons which may lead to a lack of identification:

- The lipids were not included in the database used for the identification. This was most likely the case for, e.g., NAPE and NALPE in the extracts. Both had been reported to occur in bread dough and bread, especially after lipase treatment (13). However, these are only minor compounds and their lack is not expected to influence the outcome of this study.
- The total amount in the samples and/or the ionization conditions were not optimal for some lipid classes. Cholesterol and derivatives were expected to be found in the samples due to the portion of fat originating from cow’s milk and egg, but they were not identified. In effect, the heated electrospray ionization MS-method is not suited to capture cholesterol. Nonetheless, the extraction procedure and the ionization conditions were suitable for the major apolar and polar lipid classes in the samples as TG and glycerophospholipids, respectively.
- Some lipids might have been lost during sample preparation. Especially volatile free FA did not withstand baking, freeze-drying and milling. For the analysis of free FA by lipases, more gentle procedures and gas chromatographic techniques should be used (9).
- Finally, some lipid classes might not have been found, because they were hydrolyzed and no longer present in their original form. This could be the case for ceramides, which were expected to be present due to their occurrence in milk fat. Instead of ceramides, sphingosines were found, which are formed when ceramides are hydrolyzed.

To sum up, the method covered the majority of lipid classes which were expected to occur in the samples. Some minor compounds could not be identified, probably due to their low abundance in the processed samples and/or poor ionization behavior. All major components, including those belonging to, e.g., TG, DG, PC, LPC, PE, and LPE were captured and successfully identified. With those compounds counting among the most important lipids in foodstuff in general, the method could be used for further lipidomics analyses of food. The named limitations can thereby be used as a starting point to optimize it for future research.

### 4.2 Identified lipid classes

In basic cake batter and cake, the lipids originate mostly from butter. Taking a lipid content of 2% for wheat flour (28) and 82% for butter into account, basic cake consists of 14% of fat before baking, 94% of which is from butter. Thus, a lipid distribution consisting mainly of TG was expected. More lipid classes were identified in cake than in batter. It is known from bread that baking increases the extractability of lipids, especially for lysoglycerophospholipids and glyceroglycolipids (13). This explains the identification of PE and MGDG in baked basic cake when neither had been detected in the extracts of basic cake batter. Instead of DG, MG were found after baking. When TG are hydrolyzed, DG are formed. It was therefore expected that an overall increase in DG after treatment with lipases should occur. However, a study by Schaffarczyk et al. (29) for lipase-treated bread doughs showed that the overall level of DG decreased during proofing with lipases. This indicates that rather a loss of DG takes place when samples are treated with lipases. DG are thus most likely present in low amounts. The same is true for MG, which probably also have a low abundance.

In pound cake, eggs also contribute to the total share of lipids, besides butter and wheat flour. Compared to basic cake, the samples contained more fat (24%), 13% of which originated from eggs [calculated based on an egg lipid content of 12% (28)]. Accordingly, a great variety of glycerophospholipids was expected and found in the samples. Among them were PEt, BisMePA and PIP3. Besides, also SPB were identified. Their occurrence was not expected and probably due to processing either of the ingredients themselves (e.g., pasteurization of eggs) or the sample manufacturing.

Brioche also contains eggs, albeit to a lesser extent than pound cake. Accordingly, similar lipid classes were expected and identified. Concerning lipid classes, the only difference between brioche dough and pound cake batter was the identification of PIP2 and PIP3 instead of PA. This could be due to an altered extractability caused by the yeast-based dough system of brioche compared to the yeast-free batter of pound cake. After baking, in addition to the lipid classes already present in the dough, PG were found in the baked brioche. PG are associated with cell membranes of animals, plants and microorganisms. They were identified in the lipidomes of wheat, egg, and milk (11, 17, 23). Possibly they were also present in pound cake and basic cake, but not identified. This might be due to a lower level in pound cake and basic cake compared to brioche. Besides, proofing and fermentation by yeast can affect the population of glycerophospholipids (13). Their formation during fermentation is possible and could also explain their occurrence in brioche compared to pound cake and basic cake. Additionally, their hydrolyzed form (but not PG) was found in basic cake, hinting that their degradation by lipases was more effective in basic cake.

### 4.3 Substrate specificities in relation to macroscopic effects

Lipases have preferred substrates, but commonly interact with a wide range of lipids (1). This was true for all lipases included in this study. Although labelled as a glycolipase, lipase K also interacted with other lipid classes such as TG, glycerophospholipids, and sphingolipids.

The relationship between the macroscopic effects of lipase addition in cake batter/dough as well as baked cake and the analyzed substrate specificities will be discussed in the following.

The properties of basic cake batter were improved by lipase addition (4). The lipases A, G and J were most effective at reducing batter density (G and J), stickiness (A, G, and J) and improved its rheological properties in terms of an increased linear viscoelastic region (A, G, and J) and a higher liquefaction (A, G, and J).

Concerning the substrate specificity patterns in basic cake batter, the expectation of a cluster of the lipases A, G and J based on the results from the macroscopic level were not met. Instead, only lipase K had a different substrate specificity pattern than the other lipases. This difference did not match its impact on the rheological properties of the batter (4). The substrate specificities of lipases A, G and J could not be clearly differentiated from E and M. This is in contrast to the findings for batter rheology. The lipids responsible for the different impact seem to have not been extracted or identified. Interestingly, the high specificity of all lipases towards LMW-TG is in accordance with previous findings on lipase reactions with emulsified butter, leading mostly to the release of short chain fatty acids (9). TG with short chain fatty acids seem to be more easily accessible for hydrolysis than their long chain counterparts.

In baked basic cake, all lipases led to improved short-term (24 h) texture characteristics by inhibiting the staling and causing softer products after storage (2). Additionally, the lipases reduced both resilience and cohesiveness. The lipases A, G, and J were the only lipases which were effective during long-time (96 h) storage.

In terms of substrate specificity patterns in baked basic cake, the lipases A, G, J, and M were clustered closely due to their similar substrate specificities towards DGMG and MGMG. The hydrolysis of glycoglycerolipids has been reported before to positively affect the texture of bread (6, 30). The volume of bread loafs was maximized when MGMG, DGDG and NALPE were present in the loafs (6). This points to partly similar mechanisms defining the volume of bread and the texture of basic cake. It is possible that DGMG and MGMG were also partly responsible for the effects on basic cake batter. Compared to baked basic cake, only half of the DGMG and MGMG species could be identified in batter, probably due to a poorer extractability before baking as reported for bread (13). Improved extraction techniques and targeted measurements could be used to verify this assumption. The difference between lipase M and the lipases A, G, and J is probably due to lipids which were not covered in this study.

In pound cake batter, neither density nor stickiness could be improved by the use of lipases. Still, the lipases A, G, J, and K led to a liquefaction of the batter. The greatest impact on rheological properties was caused by lipase A (4).

The substrate specificity patterns of the lipases G, J and A in pound cake batter were similar due to their hydrolysis of PC and PE. Based on these results, it is likely that lipase G, whose preferred substrate type was unknown, is also a phospholipase. This hints at different underlying mechanisms in pound cake than in basic cake for lipases to release lipids that cause rheological changes in the dough. In contrast to basic cake, lysoglycerophospholipids are positively linked to the textural impact in pound cake. Similar experiments on bread showed a negative correlation of lipase specificity towards glycerophospholipids and bread loaf volume (30), although possible synergistic effects of LPC and glyceroglycolipids have been discussed (29). Pound cake differs from bread in matter of ingredients and underlying mechanisms for baking quality. In pound cake, gas cells are mostly stabilized by polar lipids and lipoproteins while in bread, gluten and non-starch polysaccharides play a key role (31, 32). Its deviating properties require other lipids to improve its texture. However, the specificity for lysoglycerophospholipids is not the only criterion for a positive effect, as the lipase K was not clustered together with A, G, and J.

In baked pound cake, several lipases lead to textural improvements (2). The lipases A, G, and J impaired staling in the products, while A, G, J, and E all reduced the resilience and the cohesiveness considerably.

The PCA of baked pound cake separated the lipases according to their indicated type of lipase. The TG-lipase O was shown to mostly interact with TG, the phospholipases A, E, J, and M as well as the putative phospholipase G were clustered together and the glycolipase K was in between the two groups. Within the phospholipases, the lipases A, G, and J were especially close to each other, again due to their ability to release LPC and LPE. As these three were also found to improve the texture of pound cake to a similar extent, the assumption of lysoglycerophospholipids being responsible for positive effects on pound cake was supported.

The properties of brioche dough were not significantly affected by lipase addition (4). The lipase substrate specificities in brioche dough were less particular for single lipid classes. The main differences between the lipases were due to their reaction towards different LMW-TG and MMW-TG. Compared to pound cake, the same lipid classes were present for hydrolysis as similar ingredients were used. However, the specificities of all lipases did not match between the two recipes. This supports the assumption that lipase substrate specificity depends on the matrix and the accessibility of substrates. In the yeast-based dough, different mechanisms are responsible for stabilizing interfaces than in the batter-based pound cake. In this case, this seems to be the cause for a lack of textural effects by lipases on brioche dough. The total amount of lipids which were hydrolyzed is sufficient, but the type of lipids which was hydrolyzed did not lead to functional effects. Previous assumptions of a hindrance of lipase activity within brioche dough (4) to explain the missing effects are therefore less likely. Interestingly, it was stated that PC and TG in bread are only hydrolyzed during mixing and not during fermentation (33). Lipase reactions in brioche could therefore be modified by altering the mixing conditions.

The same as for brioche dough applies to baked brioche. Only small effects were caused by lipases on a textural level: The resilience and the cohesiveness of brioche were slightly reduced by the lipases E, G, and J (2). Concerning the PCA of baked brioche, the lipases E, G and J were not clustered. Instead, the reaction pattern of lipase A differed due to the specificity towards SPB. However, this property could not be linked to a textural impact of the lipase. Again, the availability of substrates for the reaction seemed to be the decisive factor for substrate specificity and concomitant effects on the texture of cakes.

## 5 Conclusion

We successfully transferred a previously established LC-MS/MS method to new and complex food matrices. The results showed that lipase substrate specificity for specific lipid classes was the reason for the improvement of batter and product quality of cakes. In the eggless basic cake, the glyceroglycolipids DGMG and MGMG were linked to macroscopic effects. In pound cake, the release of lysoglycerophospholipids such as LPC and LPE was most important. In brioche, although similar substrates as in pound cake were available for the reaction, the lipases showed different substrate specificities. This was most probably due to the accessibility of the substrates at interfaces. Differences in matrix complexity may have caused discrepancies between batter/dough and baked samples.

Our hypothesis that key compounds and reactions responsible for the suitability of lipases as baking improvers can be identified was hereby confirmed. We thus determined prerequisites for the successful application of lipases in cakes. However, the prediction of lipase reactions in complex media remains challenging. Further research is needed to include the detection of other lipids including volatile lipids and sterols, which can then be applied to analyze how the accessibility of substrates for lipase reactions can be improved. Additionally, the use of LC-MS/MS analyses for the determination of lipase substrate specificity was demonstrated. The method can be used as a starting point for establishing a common procedure for the analysis of lipids and lipase reactions in complex media like food. Future fractionation-reconstitution or addition experiments will help verify the specific effect of certain lipids on the properties of cake doughs and the products.

## Data availability statement

The original contributions presented in the study are publicly available. This data can be found here: https://dx.doi.org/10.35097/1758.

## Author contributions

CS: Conceptualization, Data curation, Formal analysis, Investigation, Methodology, Visualization, Writing – original draft. SG: Supervision, Writing – review & editing. AC: Resources, Methodology, Supervision, Writing – review & editing. KS: Funding acquisition, Project administration, Resources, Supervision, Writing – review & editing.
