# Supplementary material for: Lipidomic insights into the reaction of baking lipases in cakes
Source: Front Nutr. 2023 Dec 12;10:1290502. doi: 10.3389/fnut.2023.1290502 (PMC10773883; doi:10.3389/fnut.2023.1290502)
Supplement: Supplementary file 1 [file Data_Sheet_1.PDF]

Supplementary Material

Lipidomic insights into the reaction of baking lipases in cakes

Charlotte Dorothea Stemler[1], Sabrina Geisslitz[1], Adele Cutignano[2*], Katharina Anne Scherf[1*]

[1] Department of Bioactive and Functional Food Chemistry, Institute of Applied Biosciences, Karlsruhe Institute of Technology (KIT), Adenauerring 20 a, 76131 Karlsruhe, Germany

[2] Istituto di Chimica Biomolecolare (ICB), Consiglio Nazionale delle Ricerche (CNR), via Campi Flegrei, 34 – 80078 Pozzuoli (Napoli), Italy

*** Correspondence:**
Corresponding Authors
Katharina A. Scherf, Karlsruhe Institute of Technology (KIT), Adenauerring 20 a, 76131 Karlsruhe, Germany, Email: katharina.scherf@kit.edu, https://bioactivefc.iab.kit.edu

Adele Cutignano, Consiglio Nazionale delle Ricerche (CNR), Istituto di Chimica Biomolecolare (ICB), via Campi Flegrei, 34 - Comprensorio Olivetti, 80078 Pozzuoli (NA), Italy, Email: acutignano@icb.cnr.it

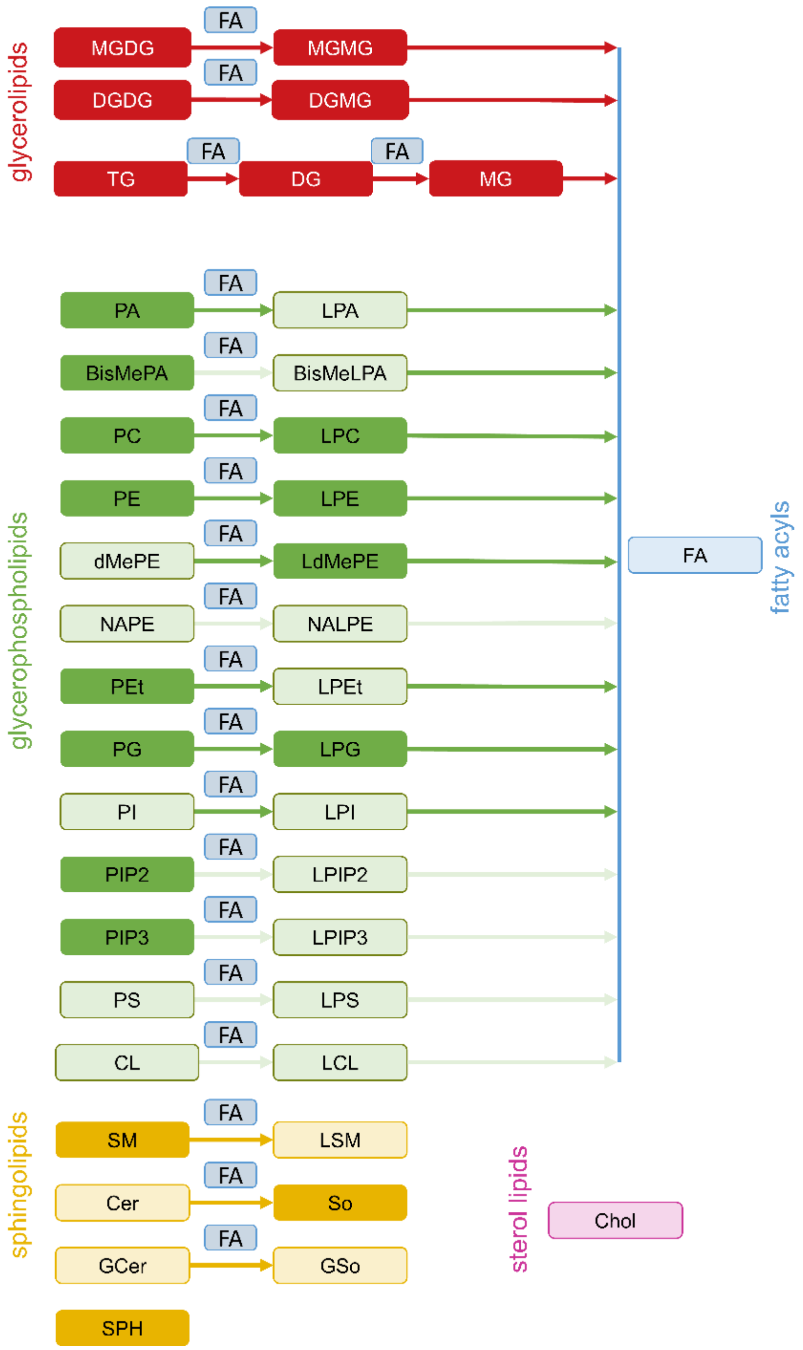

Figure S1: Expected and identified lipid classes in cake samples and their hydrolysis products. Lipids in filled frames were identified in the samples, lipids in darker frames were either expected from literature data on the ingredients or predicted as hydrolysis products. Arrows symbolize the hydrolysis as catalyzed by lipases. All abbreviations correspond to the ones used in the text. The prefix L generally indicates the lyso form of a lipid, e.g. PC glycerophosphocholine, LPC lysoglycerophosphocholine. For the complete list of abbreviations, please refer to the main text.

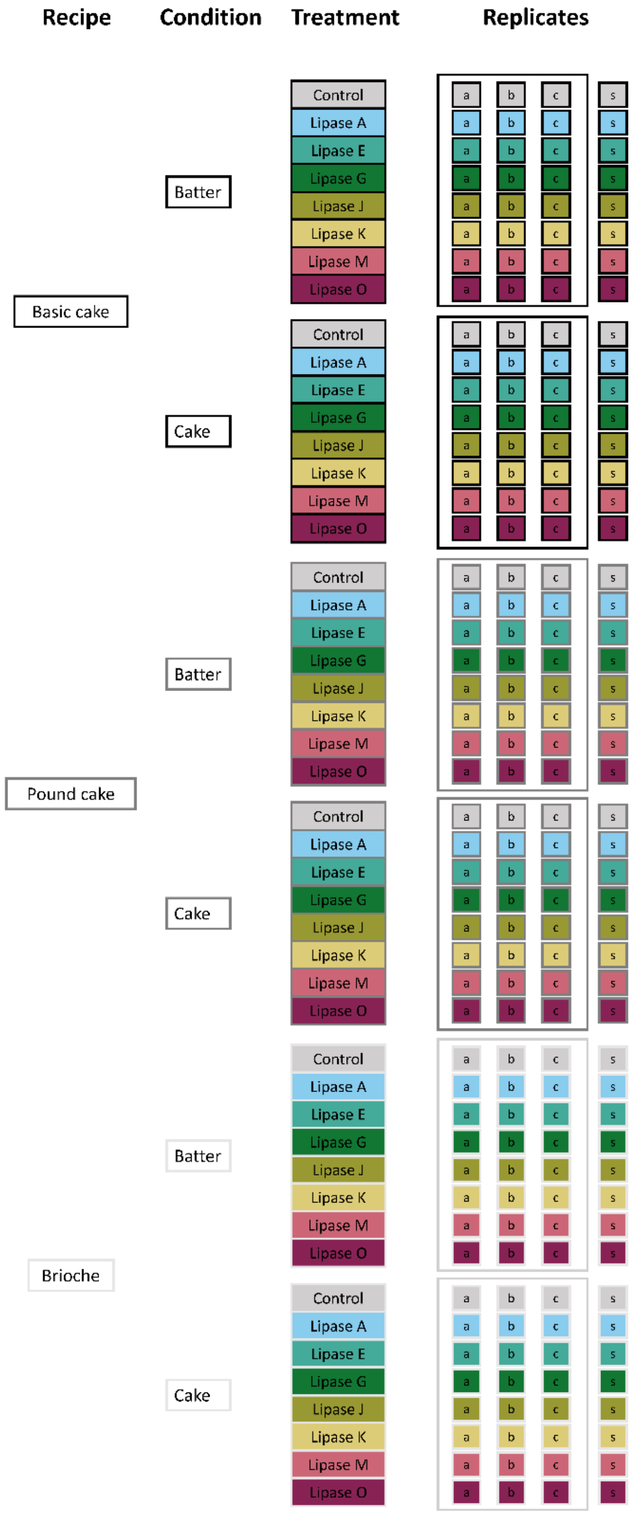

Figure S2: Schematic representation of samples. Three different recipes were prepared in two conditions with eight modifications each. For each modification, three replicates (a-c) and a spiked sample (s) were made. For quality control samples, the 24 replicates a-c of one batch (one condition of one recipe) were combined, as indicated by frames.

Table S1: Operating conditions for mass spectrometry analysis.

Samples	Basic cake			Pound cake, brioche		
System	**Infinity 1290 UHLPC System (Agilent Technologies, Santa Clara, USA)**			**Vanquish Flex Binary UHPLC (Thermo Fisher Scientific, Waltham; USA)**		
Column	Kinetex Biphenyl column (2.6 µm, 150 x 2.1 mm) (Phenomenex, Bologna, Italy)					
Column temperature	40 °C					
Flow rate	0.3 mL_min					
Solvents	A: acetonitrile_water 60:40 (v:v), 10 mmol_L ammonium formate, 0.1% formic acid					
	B: isopropanol_acetonitrile 90:10 (v:v), 2 mmol_L ammonium formate, 0.1% formic acid					
Gradient	Time [min]	A [%]	B [%]	Time [min]	A [%]	B [%]
	0.0	80	20	0.0	80	20
	4.0	60	40	4.0	60	40
	11.0	50	50	13.0	50	50
	16.0	1	99	20.0	1	99
	16.5	80	20	20.5	80	20
	21.0	80	20	25.0	80	20
Detector	Q Exactive Hybrid Quadrupole-Orbitrap (Thermo Fisher Scientific)			Q-Exactive Plus Orbitrap (Thermo Fisher Scientific)		
	Full MS + Top 10 dd MS^2 in positive and negative mode, heated electrospray ionization (HESI)					
Spray voltage	Positive 3.2 kV, negative 3.0 kV					
Capillary temperature	320 °C					
Resolution	Full MS 70,000, dd MS^2 17,500					
Auxiliary Gas	350 °C, rate 35			350 °C, rate 10		
Sheath Gas	Rate 60			Rate 35		
Scan Range	200 – 1200 *m_z*			150 - 1800 *m_z*		
Automatic gain control target	1e5			2e5		
Acquisition time	70 ms			50 ms		
Fragmentation	Stepped normalized energy 16-20 (positive), 20-40 (negative)					
Software used for evaluation	LipidSearch (Thermo Scientific, Version 4.1.30)			LipidSearch (Thermo Scientific, Version 5.0.63.7)		

Table S2: Recoveries based on TG 17:0/17:0/17:0 as internal standard.

	Basic cake						Pound cake						Brioche					
	**Batter**			**Cake**			**Batter**			**Cake**			**Dough**			**Cake**		
	a	b	c	a	b	c	a	b	c	a	b	c	a	b	c	a	b	c
**Control**	0.77	0.91	0.94	0.95	0.89	0.87	0.89	0.91	0.94	1.15	1.33	0.97	0.89	0.91	0.94	2.37	2.36	0.77
**A**	0.99	0.92	0.90	0.97	1.10	1.03	0.68	0.87	1.05	0.90	0.93	0.94	0.68	0.87	1.05	0.79	0.96	1.40
**E**	0.92	0.95	1.11	0.88	.090	.069	0.79	0.72	0.70	0.93	0.61	0.86	0.79	0.72	0.70	2.33	3.17	2.09
**G**	0.94	0.97	0.98	0.95	0.96	0.92	0.58	0.65	0.60	2.08	2.62	2.05	0.58	0.65	0.60	1.41	1.87	1.44
**J**	1.25	1.15	1.09	0.95	1.00	1.01	1.38	1.76	1.75	1.39	1.57	1.73	1.38	1.76	1.75	1.51	2.07	1.45
**K**	0.98	0.89	1.02	1.00	1.00	0.95	0.74	0.94	0.77	1.57	1.69	0.95	1.38	0.94	0.77	1.94	1.55	1.56
**M**	0.85	0.79	0.87	0.96	0.97	0.98	0.93	0.79	0.90	2.72	2.71	1.73	0.93	0.79	0.90	1.80	1.95	1.41
**O**	0.84	0.90	0.72	0.98	0.96	0.97	0.93	1.03	1.15	1.20	0.90	1.21	0.93	1.03	1.15	2.25	1.85	1.10

Table S3: Identified lipid classes and corresponding ions which were used for quantification. For the complete list of abbreviations, please refer to the main text.

**Lipid class**	**Polarity**	**Adduct**
MGDG	positive	$[M+NH_4]^+$
MGMG	negative	$[M+HCOO]^-$
DGDG	positive	$[M+NH_4]^+$
DGMG	negative	$[M+HCOO]^-$
TG	positive	$[M+NH_4]^+$
DG	positive	$[M+NH_4]^+$
MG	positive	$[M+H]^+$
PA	negative	$[M-H]^-$
BisMePA	positive	$[M+NH_4]^+$
PC	positive	$[M+H]^+$
LPC	positive	$[M+H]^+$
PE	positive	$[M+H]^+$
LPE	positive	$[M+H]^+$
LdMePE	negative	$[M-H]^-$
PEt	positive	$[M+H]^+$
PG	positive	$[M+H]^+$
LPG	negative	$[M-H]^-$
PIP2	positive	$[M+NH_4]^+$
PIP3	positive	$[M+NH_4]^+$
SM	positive	$[M+H]^+$
So	positive	$[M+H]^+$
SPB	positive	$[M+H]^+$

Table S4: Substrate specificity factors depending on the lipase (A, E, G, J, K, M or O) in basic cake batter (given as mean, n = 3).

	A	E	G	J	K	M	O
DG 18:1_12:0	6.261	4.828	5.678	4.906	0.778	4.555	11.163
DG 18:1_14:0	3.035	2.244	3.042	2.176	0.735	2.081	5.584
DG 16:0_16:0	0.776	0.778	1.044	0.661	0.800	0.712	5.797
DG 16:1_18:1	2.160	1.731	1.986	1.708	0.748	1.653	3.751
DG 16:0_18:1	1.410	1.318	1.515	1.123	0.627	1.220	3.444
DG 18:0_16:0	0.793	0.870	1.063	0.716	0.801	0.658	6.237
DG 18:1_18:2	4.969	3.120	4.094	3.893	1.332	3.062	7.826
DG 18:1_18:1	3.072	3.011	2.605	2.294	0.713	2.730	6.062
DGDG 16:0_18:2	0.073	0.140	0.049	0.061	0.527	0.246	0.757
DGDG 18:2_18:2	0.062	0.099	0.044	0.058	0.501	0.169	0.878
DGMG 18:2	3.378	3.178	2.553	3.588	3.111	3.748	1.162
LPC 16:0	0.839	0.869	0.808	0.667	0.906	1.018	0.997
LPC 18:2	0.863	0.888	0.824	0.701	0.906	1.055	0.938
MGMG 18:2	1.187	0.826	0.859	1.311	1.933	0.912	1.075
PE 18:2_18:2	0.793	0.393	0.538	0.867	1.153	0.625	0.915
So d12:0;pO	0.430	0.656	1.125	0.989	1.087	0.369	0.583
So d14:0;pO	0.484	0.633	1.000	0.700	0.937	0.398	0.518
So d16:1	0.592	0.751	0.907	0.717	0.929	1.015	1.103
So d16:0	0.578	1.049	1.252	0.634	1.050	2.440	3.535
So d16:0;pO	0.463	0.730	0.900	0.519	0.807	0.358	0.532
TG 4:0_8:0_10:0	7.709	11.360	8.180	6.953	0.976	9.222	15.140
TG 6:0_8:0_10:1	4.683	6.001	4.520	3.878	0.791	5.519	8.194
TG 4:0_10:0_10:0	1.513	2.425	1.752	1.207	0.804	1.924	3.251
TG 4:0_8:0_14:1	2.333	3.153	2.227	1.847	0.893	2.665	4.373
TG 4:0_10:0_12:0	0.504	0.741	0.510	0.416	0.836	0.763	0.921
TG 4:0_10:0_14:1	0.703	1.083	0.672	0.579	0.873	1.053	1.531
TG 4:0_10:0_14:0	0.371	0.437	0.279	0.337	0.864	0.567	0.412
TG 4:0_8:0_18:1	0.549	0.646	0.405	0.432	0.852	0.740	0.741
TG 4:0_12:0_14:0	0.408	0.394	0.250	0.372	0.891	0.582	0.321
TG 4:0_10:0_18:2	0.840	0.863	0.580	0.716	0.887	1.040	0.950
TG 4:0_10:0_18:1	0.557	0.593	0.392	0.477	0.853	0.731	0.614
TG 4:0_14:0_14:0	0.448	0.355	0.246	0.423	0.892	0.595	0.248
TG 6:0_10:0_18:2	0.742	0.689	0.508	0.626	0.873	0.853	0.696
TG 4:0_12:0_18:1	0.485	0.436	0.309	0.445	0.895	0.631	0.381
TG 4:0_14:0_16:0	0.578	0.335	0.278	0.470	0.881	0.662	0.212

TG 4:0_14:0_17:1	0.478	0.378	0.287	0.420	0.901	0.617	0.276
TG 4:0_14:0_18:2	0.583	0.360	0.286	0.498	0.897	0.656	0.237
TG 4:0_14:0_18:1	0.625	0.503	0.389	0.510	0.858	0.697	0.410
TG 4:0_16:0_16:0	0.469	0.363	0.250	0.418	0.884	0.569	0.250
TG 4:0_15:0_18:1	0.662	0.393	0.354	0.589	0.942	0.775	0.269
TG 4:0_16:0_17:1	0.459	0.302	0.238	0.438	0.921	0.575	0.205
TG 4:0_16:0_17:0	0.649	0.386	0.344	0.522	0.912	0.700	0.247
TG 4:0_16:0_18:2	0.549	0.416	0.316	0.484	0.927	0.658	0.297
TG 4:0_16:0_18:1	0.469	0.324	0.241	0.398	0.906	0.556	0.184
TG 6:0_16:0_16:0	0.775	0.421	0.406	0.615	0.916	0.752	0.291
TG 4:0_17:1_18:1	0.553	0.417	0.302	0.445	0.871	0.622	0.304
TG 4:0_17:0_18:1	0.532	0.347	0.289	0.503	0.923	0.621	0.216
TG 6:0_16:0_17:1	0.544	0.303	0.276	0.483	0.887	0.622	0.177
TG 6:0_16:0_17:0	0.745	0.429	0.410	0.612	0.962	0.772	0.302
TG 4:0_18:1_18:1	0.559	0.390	0.302	0.490	0.853	0.613	0.302
TG 6:0_16:0_18:1	0.575	0.352	0.302	0.520	0.957	0.670	0.212
TG 4:0_16:0_20:0	0.995	0.674	0.597	1.057	1.516	1.362	0.530
TG 16:0_10:0_14:0	0.758	0.448	0.454	0.645	0.911	0.768	0.347
TG 6:0_17:1_18:1	0.609	0.433	0.363	0.550	0.947	0.736	0.389
TG 6:0_18:1_18:1	0.617	0.426	0.380	0.559	0.909	0.660	0.405
TG 16:0_8:0_18:1	0.668	0.391	0.414	0.612	0.975	0.709	0.322
TG 16:0_10:0_16:0	0.841	0.557	0.562	0.676	0.910	0.859	0.527
TG 8:0_17:1_18:1	0.660	0.451	0.430	0.576	0.909	0.696	0.487
TG 15:0_10:0_18:1	0.743	0.453	0.473	0.628	0.922	0.721	0.419
TG 15:0_12:0_16:0	0.819	0.585	0.598	0.671	0.936	0.849	0.603
TG 8:0_18:1_18:1	0.696	0.449	0.454	0.581	0.902	0.691	0.471
TG 16:0_10:0_18:1	0.702	0.447	0.451	0.605	0.911	0.733	0.374
TG 16:0_12:0_16:0	0.886	0.694	0.625	0.688	0.919	0.835	0.686
TG 10:0_17:1_18:1	0.721	0.526	0.483	0.620	0.912	0.744	0.599
TG 10:0_18:1_18:1	0.722	0.503	0.514	0.624	0.948	0.743	0.562
TG 16:0_12:0_18:1	0.800	0.544	0.585	0.683	0.998	0.896	0.509
TG 16:0_14:0_16:0	0.885	0.809	0.765	0.749	1.005	0.995	0.870
TG 18:1_12:0_18:2	0.782	0.585	0.571	0.670	0.969	0.819	0.640
TG 16:0_14:1_18:1	0.788	0.518	0.527	0.636	0.917	0.783	0.555
TG 16:0_14:0_18:1	0.829	0.548	0.590	0.685	0.936	0.809	0.539
TG 16:0_16:0_16:0	0.883	0.810	0.757	0.748	0.995	0.903	0.836
TG 16:0_16:0_18:1	0.856	0.631	0.660	0.712	0.901	0.891	0.605

TG 18:0_16:0_16:0	0.895	0.783	0.775	0.768	0.959	1.056	0.855
TG 18:1_14:0_18:2	0.794	0.596	0.569	0.678	0.955	0.792	0.623
TG 18:1_14:0_18:1	0.844	0.575	0.548	0.643	0.891	0.797	0.516
TG 16:0_17:0_18:1	0.853	0.636	0.647	0.723	0.946	0.908	0.604
TG 16:0_18:1_18:2	0.747	0.547	0.550	0.662	0.926	0.815	0.530
TG 16:0_18:1_18:1	0.754	0.512	0.518	0.666	0.887	0.834	0.454
TG 18:0_16:0_18:1	0.785	0.571	0.576	0.669	0.819	0.866	0.539
TG 18:0_16:0_18:0	0.800	0.707	0.702	0.709	0.879	0.868	0.734
TG 16:0_18:1_18:3	0.723	0.584	0.592	0.796	0.844	0.782	0.585
TG 17:0_18:1_18:1	0.720	0.465	0.498	0.676	0.890	0.835	0.436
TG 18:0_17:0_18:1	0.809	0.561	0.586	0.688	0.905	0.926	0.537
TG 18:1_18:1_18:2	0.712	0.532	0.532	0.664	0.875	0.818	0.644
TG 18:1_18:1_18:1	0.775	0.500	0.503	0.681	0.924	0.843	0.552
TG 18:0_18:1_18:1	0.782	0.484	0.481	0.734	0.978	0.850	0.389
TG 18:0_18:0_18:1	0.846	0.590	0.567	0.792	1.003	0.984	0.517
TG 18:0_18:0_18:0	0.827	0.720	0.637	0.698	0.905	0.955	0.720
TG 18:2_18:2_18:2	0.592	0.412	0.453	0.497	0.773	0.602	0.360
TG 19:1_18:1_18:1	0.745	0.515	0.487	0.659	0.882	0.774	0.520
TG 19:0_18:1_18:1	0.765	0.517	0.492	0.618	0.833	0.744	0.405
TG 16:0_16:0_23:0	0.836	0.745	0.716	0.671	0.908	0.906	0.817
TG 20:1_18:1_18:1	0.797	0.586	0.553	0.681	0.966	0.810	0.524
TG 20:0_18:1_18:1	0.769	0.517	0.517	0.641	0.966	0.758	0.437
TG 16:0_18:1_22:0	0.852	0.623	0.604	0.686	0.924	0.892	0.608
TG 16:0_16:0_24:0	0.859	0.734	0.707	0.697	0.901	0.937	0.820
TG 16:0_18:1_23:1	0.842	0.560	0.588	0.704	0.942	0.846	0.500
TG 18:0_16:0_23:1	0.876	0.692	0.725	0.719	0.960	0.919	0.691
TG 18:1_18:1_22:0	0.792	0.581	0.615	0.693	0.937	0.856	0.501
TG 16:0_18:1_24:0	0.815	0.625	0.648	0.705	0.897	0.877	0.606
TG 26:0_16:0_16:0	0.901	0.828	0.802	0.731	0.944	0.975	0.838
TG 25:1_16:0_18:1	0.808	0.602	0.623	0.710	0.958	0.880	0.551
TG 18:1_18:1_24:0	0.798	0.604	0.627	0.677	0.935	0.860	0.542
TG 26:0_16:0_18:1	0.822	0.674	0.664	0.722	0.939	0.897	0.632
TG 26:0_16:0_18:1	0.859	0.697	0.685	0.738	0.950	0.901	0.625

Table S5: Substrate specificity factors depending on the lipase (A, E, G, J, K, M or O) in basic cake (given as mean, n = 3).

	A	E	G	J	K	M	O
DGDG 16:0_18:2	0.149	0.820	0.214	0.191	1.174	0.914	1.386
DGDG 18:2_18:3	0.145	0.819	0.171	0.149	1.244	0.918	1.442
DGDG 18:2_18:2	0.122	0.813	0.170	0.159	1.176	0.879	1.390
DGDG 18:1_18:2	0.184	0.839	0.244	0.213	1.148	0.895	1.388
DGMG 18:3	26.437	4.166	39.155	36.602	4.339	8.321	2.200
DGMG 18:2	13.242	2.613	18.783	17.551	2.335	3.884	1.834
LPC 16:0	3.126	1.129	4.987	3.456	1.363	1.384	1.603
LPC 18:3	2.121	1.165	3.308	2.713	1.330	1.318	1.539
LPC 18:2	2.304	1.146	3.597	2.868	1.311	1.299	1.523
LPE 16:0	4.644	1.191	7.328	4.953	1.828	1.788	1.825
LPG 16:0	2.709	1.215	3.607	3.213	1.394	1.584	1.538
LdMePE 18:2	2.653	1.065	4.482	3.450	1.586	1.471	1.663
MG 24:2	0.708	1.177	1.189	1.481	1.710	1.680	1.483
MGDG 16:0_18:2	0.404	0.994	0.679	0.594	1.419	1.232	1.467
MGDG 18:2_18:2	0.071	0.435	0.103	0.070	0.950	0.467	1.367
MGDG 18:1_18:2	0.168	0.545	0.313	0.236	0.957	0.630	1.312
MGMG 18:2	7.550	2.511	11.799	10.628	2.689	3.670	1.818
PC 22:0_12:2	0.267	0.682	0.305	0.245	1.012	0.691	1.311
PE 18:2_18:2	2.117	2.138	3.430	3.407	2.100	2.510	1.488
PE 18:1_18:2	2.173	2.092	3.501	3.555	2.103	2.511	1.492
So d12:0;pO	1.566	3.133	1.222	1.909	1.475	1.346	3.294
So d14:0;pO	1.565	2.257	1.138	1.752	1.744	0.961	2.519
So d16:1	1.556	1.727	1.368	1.680	1.563	0.937	1.739
So d16:0	1.727	1.704	0.927	1.680	1.653	0.661	1.627
So d16:0;pO	1.196	1.320	0.730	1.019	1.584	0.770	1.829
TG 4:0_8:0_10:0	3.002	1.007	4.520	2.719	1.351	1.306	1.467
TG 4:0_8:0_12:0	0.914	1.041	1.377	1.091	1.321	1.321	1.125
TG 4:0_10:1_12:0	0.753	1.065	1.117	1.058	1.318	1.296	1.239
TG 4:0_10:0_12:0	0.543	1.153	0.791	0.895	1.374	1.342	1.187
TG 4:0_8:0_15:0	0.485	1.150	0.744	0.889	1.350	1.339	1.172
TG 4:0_8:0_16:1	0.540	1.145	0.838	0.992	1.425	1.402	1.207
TG 4:0_10:0_14:0	0.505	1.157	0.774	0.927	1.418	1.390	1.210
TG 4:0_10:0_15:0	0.506	1.132	0.775	0.976	1.387	1.359	1.194
TG 4:0_8:0_18:1	0.514	1.122	0.811	1.005	1.419	1.397	1.195

TG 4:0_10:0_16:0	0.511	1.096	0.801	0.993	1.467	1.398	1.212
TG 4:0_10:0_17:1	0.490	1.115	0.883	1.029	1.514	1.410	1.169
TG 4:0_12:0_15:0	0.510	1.079	0.808	0.992	1.474	1.415	1.244
TG 4:0_10:0_18:1	0.587	1.144	0.898	1.093	1.530	1.447	1.261
TG 4:0_12:0_16:0	0.542	1.106	0.841	1.052	1.537	1.450	1.283
TG 4:0_12:0_17:1	0.534	1.113	0.859	1.078	1.604	1.449	1.311
TG 4:0_14:0_15:0	0.525	1.112	0.826	1.034	1.480	1.421	1.264
TG 4:0_12:0_18:2	0.706	1.140	1.150	1.311	1.617	1.492	1.298
TG 6:0_10:0_18:1	0.551	1.072	0.876	1.081	1.455	1.437	1.250
TG 4:0_14:0_17:1	0.538	1.076	0.813	1.052	1.527	1.385	1.237
TG 4:0_15:0_16:0	0.552	1.114	0.908	1.155	1.561	1.420	1.265
TG 4:0_14:0_18:2	0.538	1.119	0.845	1.096	1.551	1.498	1.307
TG 4:0_14:0_18:1	0.638	1.085	1.022	1.214	1.548	1.419	1.247
TG 4:0_16:0_16:0	0.575	1.111	0.904	1.134	1.562	1.539	1.294
TG 4:0_15:0_18:2	0.525	1.105	0.916	1.145	1.688	1.574	1.371
TG 4:0_15:0_18:1	0.608	1.214	1.115	1.262	1.648	1.658	1.373
TG 4:0_16:0_17:1	0.534	1.084	0.944	1.146	1.638	1.535	1.333
TG 6:0_15:0_16:0	0.515	1.108	0.917	1.162	1.777	1.569	1.366
TG 4:0_16:1_18:1	0.583	1.104	0.951	1.200	1.631	1.514	1.293
TG 4:0_16:0_18:1	0.590	1.096	1.027	1.213	1.649	1.557	1.342
TG 4:0_14:0_20:0	0.575	1.100	0.956	1.203	1.730	1.586	1.307
TG 6:0_16:0_16:0	0.658	1.314	1.056	1.493	1.849	1.656	2.507
TG 4:0_17:1_18:1	0.586	1.120	1.051	1.335	1.733	1.573	1.423
TG 4:0_17:0_18:1	0.597	1.061	0.987	1.269	1.739	1.633	1.330
TG 6:0_16:0_17:1	0.614	1.135	1.093	1.342	1.706	1.572	1.400
TG 6:0_16:0_17:0	0.596	1.063	0.912	1.352	1.646	1.621	1.460
TG 4:0_18:1_18:2	0.619	1.061	1.034	1.302	1.615	1.529	1.357
TG 4:0_18:1_18:1	0.635	1.184	1.130	1.358	1.761	1.590	1.259
TG 6:0_16:0_18:1	0.592	1.057	1.029	1.282	1.648	1.540	1.353
TG 4:0_16:0_20:0	0.622	1.080	1.046	1.338	1.716	1.620	1.393
TG 16:0_10:0_14:0	0.732	1.381	1.115	1.601	1.801	1.618	2.493
TG 6:0_17:1_18:1	0.642	1.140	1.199	1.474	1.747	1.709	1.466
TG 6:0_17:0_18:1	0.641	1.101	1.090	1.440	1.727	1.608	1.395
TG 16:0_8:0_17:1	0.638	1.123	1.201	1.483	1.760	1.698	1.427
TG 15:0_10:0_16:0	0.655	0.979	1.032	1.277	1.589	1.605	1.507
TG 6:0_18:1_18:1	0.703	1.134	1.206	1.528	1.689	1.635	1.422
TG 4:0_18:1_20:0	0.663	1.116	1.209	1.504	1.730	1.697	1.376
TG 16:0_8:0_18:1	0.667	1.218	1.084	1.525	1.722	1.622	2.303

TG 6:0_16:0_20:0	0.753	1.286	1.277	1.611	1.793	1.719	1.554
TG 16:0_10:0_16:0	0.686	1.291	1.217	1.534	1.603	1.585	2.086
TG 8:0_17:1_18:1	0.727	1.104	1.239	1.418	1.613	1.569	1.442
TG 15:0_10:0_18:1	0.758	1.184	1.241	1.547	1.792	1.666	1.540
TG 16:0_10:0_17:1	0.711	1.154	1.261	1.450	1.637	1.563	1.414
TG 15:0_12:0_16:0	0.723	0.994	1.209	1.405	1.578	1.564	1.789
TG 8:0_18:1_18:1	0.748	1.165	1.270	1.485	1.587	1.497	1.417
TG 16:0_10:0_18:1	0.709	1.157	1.230	1.476	1.648	1.551	1.386
TG 20:0_8:0_16:0	0.726	1.109	1.228	1.416	1.566	1.458	1.385
TG 16:0_12:0_16:0	0.694	1.223	1.208	1.417	1.589	1.497	2.176
TG 10:0_17:1_18:1	0.812	1.159	1.352	1.459	1.492	1.492	1.421
TG 15:0_12:0_18:1	0.781	1.252	1.329	1.504	1.647	1.541	1.437
TG 15:0_14:0_16:0	0.791	1.169	1.345	1.504	1.582	1.555	1.463
TG 10:0_18:1_18:1	0.825	1.099	1.288	1.506	1.496	1.507	1.433
TG 16:0_12:0_18:1	0.779	1.124	1.305	1.532	1.589	1.571	1.421
TG 20:0_10:0_16:0	0.797	1.046	1.211	1.403	1.495	1.438	1.408
TG 16:0_14:0_16:0	0.824	1.456	1.381	1.793	1.584	1.650	2.235
TG 11:0_18:1_18:1	0.857	1.186	1.325	1.449	1.430	1.376	1.412
TG 15:0_14:0_18:1	0.796	1.156	1.231	1.432	1.476	1.380	1.408
TG 16:0_12:0_19:0	0.780	1.149	1.238	1.392	1.433	1.346	1.356
TG 15:0_16:0_16:0	0.902	1.375	1.371	1.924	1.722	1.721	2.078
TG 16:0_14:1_18:1	0.762	1.143	1.257	1.433	1.498	1.395	1.383
TG 20:0_10:0_18:1	0.815	1.184	1.292	1.418	1.495	1.397	1.368
TG 16:0_14:0_18:1	0.952	1.310	1.348	1.658	1.680	1.627	2.222
TG 20:0_14:0_14:0	0.806	1.105	1.253	1.375	1.371	1.324	1.349
TG 16:0_16:0_16:0	0.858	1.409	1.249	1.534	1.467	1.332	1.480
TG 18:0_15:0_16:0	0.870	1.057	1.261	1.251	1.287	1.271	1.268
TG 14:0_17:1_18:2	0.874	1.073	1.255	1.329	1.182	1.223	1.278
TG 15:0_16:1_18:1	0.822	1.152	1.212	1.388	1.435	1.315	1.332
TG 15:0_16:0_18:1	0.801	1.089	1.215	1.286	1.336	1.271	1.244
TG 16:0_16:0_18:1	0.808	1.077	1.190	1.284	1.257	1.235	1.207
TG 18:0_16:0_16:0	0.816	1.164	1.256	1.312	1.249	1.230	1.296
TG 18:1_14:0_18:2	0.881	1.150	1.313	0.998	1.240	1.239	1.288
TG 18:1_14:0_18:1	0.803	1.091	1.253	1.321	1.329	1.294	1.241
TG 16:0_17:1_18:1	0.815	1.146	1.245	1.339	1.269	1.279	1.321
TG 16:0_17:0_18:1	0.806	1.144	1.235	1.334	1.292	1.284	1.275
TG 15:0_18:1_18:2	1.386	1.357	1.167	1.167	0.921	0.912	1.265

TG 16:0_18:1_18:2	0.793	1.156	1.239	1.326	1.331	1.225	1.278
TG 16:0_18:1_18:1	0.835	1.156	1.222	1.329	1.331	1.307	1.282
TG 18:0_16:0_18:1	0.865	1.157	1.241	1.363	1.306	1.243	1.355
TG 18:0_16:0_18:0	0.814	1.106	1.159	1.316	1.258	1.217	1.339
TG 16:1_18:1_18:2	0.898	1.157	1.212	1.371	1.292	1.247	1.388
TG 18:1_17:1_18:1	0.876	1.172	1.242	1.268	1.241	1.212	1.277
TG 17:0_18:1_18:1	0.847	1.104	1.210	1.390	1.327	1.273	1.364
TG 18:0_17:0_18:1	0.826	1.213	1.201	1.330	1.373	1.239	1.379
TG 18:1_18:1_18:2	1.365	1.585	1.472	1.635	1.189	1.279	1.925
TG 18:1_18:1_18:1	0.829	1.203	1.192	1.378	1.384	1.237	1.326
TG 18:0_18:1_18:1	0.778	1.127	1.160	1.329	1.419	1.229	1.321
TG 18:0_18:0_18:1	0.749	1.061	1.146	1.320	1.341	1.228	1.347
TG 18:0_18:0_18:0	0.815	1.147	1.241	1.426	1.377	1.319	1.377
TG 18:2_18:2_18:2	0.847	1.067	1.190	1.377	1.294	1.304	1.337
TG 19:1_18:1_18:1	0.803	1.136	1.201	1.242	1.308	1.171	1.181
TG 19:1_18:0_18:1	0.742	1.019	1.134	1.322	1.346	1.270	1.380
TG 16:0_16:0_23:1	0.787	1.089	1.209	1.439	1.434	1.374	1.411
TG 18:0_16:0_21:0	1.783	1.331	1.514	1.747	1.779	1.676	1.677
TG 20:1_18:1_18:1	1.768	1.291	1.505	1.728	1.725	1.644	1.805
TG 20:0_18:1_18:1	1.625	1.254	1.373	1.741	1.552	1.684	1.649
TG 16:0_18:1_22:0	1.739	1.299	1.481	1.684	1.968	1.773	1.759
TG 18:0_16:0_22:0	1.737	1.281	1.470	1.819	1.805	1.711	1.711
TG 18:1_18:1_21:1	1.937	1.332	1.645	1.901	1.849	1.873	1.897
TG 16:0_18:1_23:1	1.777	1.318	1.506	1.819	1.888	1.826	1.768
TG 16:0_18:1_23:0	1.762	1.332	1.493	1.721	1.723	1.735	1.587
TG 18:1_18:1_22:1	1.760	1.327	1.488	1.691	1.742	1.745	1.698
TG 18:1_18:1_22:0	1.766	1.349	1.509	1.705	1.897	1.870	1.809
TG 16:0_18:1_24:0	1.666	1.297	1.411	1.683	1.751	1.773	1.691
TG 18:0_16:0_24:0	1.745	1.323	1.484	1.670	1.833	1.841	1.745
TG 18:1_18:1_23:1	1.736	1.325	1.473	1.698	1.718	1.791	1.683
TG 18:0_18:1_23:1	1.691	1.328	1.434	1.759	1.704	1.726	1.651
TG 18:1_18:1_24:0	1.650	1.291	1.396	1.623	1.797	1.772	1.584
TG 26:0_16:0_18:1	1.736	1.299	1.474	1.780	1.914	1.838	1.707
TG 26:0_18:1_18:1	1.699	1.281	1.445	1.546	1.970	1.809	1.722

Table S6: Substrate specificity factors depending on the lipase (A, E, G, J, K, M or O) in pound cake batter (given as mean, n = 3).

	A	E	G	J	K	M	O
BisMePA 34:3	0.175	0.563	0.294	0.115	0.659	0.356	0.797
BisMePA 34:2	0.225	0.715	0.330	0.162	0.752	0.498	0.776
BisMePA 36:4	0.296	0.739	0.431	0.177	0.716	0.496	0.787
BisMePA 36:3	0.137	0.482	0.233	0.099	0.703	0.375	0.711
BisMePA 36:2	0.312	0.791	0.629	0.224	1.163	0.677	1.000
BisMePA 38:7	0.146	0.511	0.208	0.101	0.708	0.392	0.764
BisMePA 40:7	0.117	0.494	0.230	0.101	0.725	0.396	0.719
DG 42:6; O	1.730	1.335	1.385	0.502	0.805	0.802	0.864
DG 18:1_18:3	0.638	1.291	1.090	0.381	0.835	0.593	1.031
DG 18:2_18:2	3.579	3.003	3.064	0.928	0.900	0.929	2.269
DG 18:1_18:2	4.717	5.179	5.483	1.810	1.236	1.302	3.280
DG 16:0_24:6	0.202	0.212	0.254	0.048	0.075	0.080	0.153
DG 24:6_18:0	0.821	0.799	0.998	0.303	0.573	0.586	0.641
DGDG 16:0_18:2	0.306	0.814	0.496	0.169	0.784	0.526	0.818
DGDG 18:2_18:2	0.189	0.476	0.378	0.108	0.628	0.390	0.754
DGMG 18:2	6.585	7.891	8.927	3.575	3.213	4.001	0.935
LPC 14:0	6.459	6.920	7.262	1.693	2.010	2.850	0.681
LPC 16:1	15.105	12.757	18.202	5.537	2.871	4.584	0.645
LPC 16:0	1.180	1.461	1.453	0.474	0.911	0.785	0.690
LPC 17:1	18.678	18.533	22.115	7.145	3.667	5.933	0.715
LPC 18:3	2.170	2.243	2.048	0.533	0.932	0.935	0.469
LPC 18:2	17.371	15.394	20.753	6.291	3.562	5.408	0.653
LPC 18:1	57.785	35.009	67.293	20.660	8.285	14.998	0.642
LPC 18:0	0.987	1.269	1.236	0.379	0.809	0.665	0.698
LPC 19:1	16.506	13.555	21.196	6.071	3.444	4.352	0.720
LPC 20:4	81.739	56.360	87.726	24.743	11.790	21.170	0.545
LPC 20:1	5.303	6.657	7.421	3.367	1.846	2.674	0.686
LPC 22:6	120.099	100.788	135.303	42.267	19.837	34.251	0.702
LPC 22:5	101.030	92.024	112.959	35.789	15.694	29.041	0.643
LPE 16:0	0.899	1.339	1.460	0.440	0.972	0.685	0.775
LPE 18:2	15.943	15.402	21.633	7.035	4.174	6.288	0.752
LPE 18:1	24.263	23.020	34.504	10.825	5.964	9.484	0.821
LPE 18:0	0.985	1.325	1.395	0.501	1.031	0.733	0.837
LPE 20:4	76.807	72.833	96.542	30.373	15.751	25.572	0.888

LPE 22:6	53.615	49.646	68.778	21.970	11.536	19.272	0.641
LPE 22:5	51.765	48.248	56.756	19.434	11.437	18.126	0.688
MG 18:2	1.932	1.884	1.976	0.799	0.969	0.933	0.580
MG 17:3; O	21.957	5.597	6.279	3.254	4.642	14.296	4.024
MGDG 18:2_18:2	0.081	0.079	0.142	0.036	0.316	0.112	0.270
MGMG 18:2	3.479	3.328	5.015	1.939	2.640	2.001	1.387
PA 22:5_18:1	0.201	0.908	0.411	0.177	0.862	0.564	0.920
PC 39:10; P	0.154	0.497	0.180	0.076	0.599	0.336	0.602
PC 34:2; O	1.079	2.283	1.989	0.795	1.352	1.010	0.401
PC 39:10; O	0.236	0.529	0.314	0.137	0.800	0.461	0.632
PC 30:2	33.919	58.451	37.455	9.186	5.630	12.911	0.811
PC 30:1	18.379	43.547	20.554	5.684	3.409	5.777	0.153
PC 30:0	0.482	1.375	1.265	0.491	0.760	0.379	0.771
PC 32:3	1.171	2.753	1.549	0.569	0.742	0.747	0.600
PC 32:2	1.238	3.314	1.712	0.498	0.876	0.841	0.665
PC 32:1	0.389	0.825	0.502	0.175	0.717	0.440	0.689
PC 32:0	0.741	1.345	1.087	0.399	0.807	0.701	0.771
PC 33:2	0.269	0.839	0.228	0.125	0.795	0.652	1.100
PC 33:1	0.418	0.885	0.547	0.152	0.531	0.364	0.665
PC 34:4	0.464	1.080	0.527	0.192	0.640	0.420	0.600
PC 34:3	0.286	0.607	0.284	0.148	0.684	0.499	0.621
PC 16:0_18:2	0.164	0.361	0.159	0.071	0.448	0.312	0.791
PC 16:0_18:1	0.112	0.531	0.245	0.106	0.737	0.320	0.665
PC 34:0	0.825	1.271	1.045	0.375	0.775	0.564	0.526
PC 35:2	0.246	0.729	0.443	0.146	0.698	0.466	0.694
PC 35:1	0.360	0.837	0.455	0.203	0.771	0.487	0.686
PC 36:6	0.396	1.100	0.531	0.182	0.825	0.473	0.776
PC 36:5	0.159	0.488	0.220	0.096	0.624	0.445	0.595
PC 16:0_20:4	0.139	0.436	0.137	0.050	0.597	0.270	0.538
PC 18:1_18:2	0.319	0.941	0.460	0.188	0.697	0.507	0.682
PC 18:0_18:2	0.208	0.668	0.402	0.147	0.689	0.463	0.639
PC 18:0_18:1	0.331	0.832	0.454	0.190	0.809	0.507	0.719
PC 38:7	0.161	0.446	0.207	0.094	0.593	0.253	0.386
PC 16:0_22:6	0.175	0.669	0.343	0.135	0.873	0.420	1.061
PC 21:3_17:2	0.119	0.525	0.299	0.127	0.384	0.316	0.690
PC 18:0_20:4	0.157	0.402	0.342	0.130	0.551	0.461	0.557
PC 40:9	0.163	0.565	0.226	0.092	0.736	0.376	0.621
PC 40:8	0.280	0.853	0.356	0.167	0.534	0.339	0.479

PC 40:7	0.311	0.882	0.429	0.184	0.695	0.454	0.625
PC 40:6	0.213	0.721	0.325	0.143	0.561	0.461	0.661
PC 40:5	0.185	0.618	0.314	0.121	0.726	0.418	0.716
PE 16:0_18:2	0.172	0.549	0.305	0.113	0.776	0.425	0.772
PE 16:0_18:1	0.169	0.590	0.372	0.162	0.855	0.533	0.785
PE 18:2_18:2	1.821	2.464	3.141	0.904	1.298	1.394	0.699
PE 18:2_18:2	1.733	1.776	2.420	0.882	1.012	1.070	0.779
PE 20:4_16:0	0.213	0.621	0.350	0.129	0.632	0.415	0.770
PE 18:0_18:2	0.145	0.597	0.283	0.139	0.828	0.537	0.966
PE 18:0_18:1	0.134	0.627	0.368	0.181	0.859	0.533	0.856
PE 16:0_22:6	0.153	0.556	0.262	0.114	0.747	0.441	0.776
PE 20:4_18:1	0.200	0.790	0.357	0.154	0.743	0.486	0.793
PE 18:0_20:4	0.119	0.400	0.238	0.106	0.656	0.348	0.702
PEt 10:0_12:1	0.484	2.805	1.219	0.430	0.861	0.808	2.339
PEt 10:0_18:2	1.218	1.842	1.500	0.583	0.960	0.734	1.102
PEt 10:0_18:1	1.061	1.195	1.341	0.511	0.856	0.618	0.727
PEt 10:0_22:1	1.047	1.249	1.380	0.546	0.963	0.810	0.687
PEt 10:0_24:1	1.279	1.307	1.581	0.600	1.049	0.885	0.794
SM d32:1	1.205	1.363	1.815	0.608	1.059	0.917	0.804
SM d34:1	0.813	1.509	0.838	0.360	0.622	0.497	0.699
SM d34:0	0.855	1.226	1.191	0.431	0.772	0.607	0.587
SM d36:4	0.905	0.991	0.332	0.295	0.444	0.153	0.392
SM d36:1	1.039	1.413	1.445	0.512	0.861	0.681	0.676
SPB d14:0	2.291	1.216	1.275	0.451	0.955	0.973	0.890
SPB d18:0	1.786	0.963	0.647	0.250	0.544	0.595	0.416
SPB d14:1	2.285	0.625	0.593	0.214	0.484	0.576	0.842
SPB d16:1	1.958	0.558	0.529	0.197	0.478	0.508	0.574
TG 4:0_6:0_8:0	0.021	0.090	0.015	0.005	0.004	0.022	0.120
TG 4:0_6:0_10:0	6.126	29.661	4.903	1.324	1.484	6.428	37.416
TG 4:0_4:0_14:1	1.838	3.520	1.662	0.751	1.154	1.129	2.832
TG 4:0_6:0_12:0	1.748	7.060	1.739	0.594	0.917	1.719	8.590
TG 4:0_6:0_14:1	1.497	8.163	2.041	0.665	1.179	1.310	7.434
TG 4:0_6:0_14:0	0.813	2.117	1.146	0.337	0.569	0.439	2.246
TG 4:0_4:0_18:2	1.504	5.344	1.455	0.479	0.666	1.180	4.994
TG 4:0_4:0_18:1	1.294	3.218	1.554	0.362	0.663	0.846	3.008
TG 4:0_6:0_16:0	0.630	0.925	0.781	0.287	0.604	0.242	0.776
TG 4:0_6:0_17:1	0.876	1.932	1.099	0.401	0.901	0.638	1.717

TG 4:0_6:0_18:2	0.888	3.001	1.142	0.524	0.947	0.919	2.079
TG 4:0_6:0_18:1	0.911	1.526	1.223	0.436	0.914	0.381	0.849
TG 4:0_8:0_16:0	0.995	0.899	0.724	0.467	0.613	0.608	0.617
TG 4:0_8:0_17:1	0.799	1.335	1.189	0.404	0.685	0.502	0.777
TG 4:0_8:0_18:2	1.180	2.357	0.952	0.427	1.116	0.716	1.233
TG 4:0_8:0_18:1	1.244	1.330	1.727	0.622	1.019	0.810	0.775
TG 4:0_10:0_16:0	0.845	1.301	1.307	0.774	0.950	0.820	0.812
TG 4:0_10:0_18:2	1.209	2.135	2.218	0.617	1.368	0.948	1.279
TG 4:0_10:0_18:1	1.226	0.974	1.510	0.700	1.241	0.701	1.145
TG 4:0_14:0_14:1	0.873	1.128	1.320	0.589	1.100	0.812	1.267
TG 4:0_12:0_16:0	0.957	0.571	1.282	0.518	1.067	0.768	0.571
TG 4:0_12:0_16:0	0.841	0.674	1.057	0.578	1.037	0.470	0.500
TG 4:0_11:0_18:1	1.383	1.700	1.264	0.243	0.898	0.629	0.712
TG 4:0_15:0_14:1	0.865	1.109	1.355	0.549	0.975	0.770	0.496
TG 8:0_11:0_14:0	0.971	1.021	1.263	0.510	0.821	0.643	0.610
TG 4:0_12:0_18:2	1.097	1.615	1.324	0.463	0.966	0.700	0.770
TG 4:0_12:0_18:1	0.988	1.223	1.519	0.575	1.016	0.734	0.756
TG 4:0_14:0_16:0	1.245	1.364	1.759	0.632	1.145	0.845	0.748
TG 4:0_14:0_16:0	1.752	1.966	2.476	0.976	1.751	1.402	1.625
TG 4:0_15:0_16:1	1.064	1.148	0.850	0.586	0.618	0.617	0.538
TG 6:0_14:0_15:1	1.152	1.519	1.454	0.620	1.095	0.808	0.877
TG 4:0_14:0_18:3	1.108	1.250	1.086	0.256	0.655	0.412	0.624
TG 8:0_10:0_18:2	0.863	2.333	1.399	0.501	1.077	0.921	1.105
TG 4:0_14:0_18:1	1.032	0.739	0.142	0.361	0.064	0.056	0.446
TG 4:0_14:0_18:1	1.055	1.077	1.586	0.590	1.059	0.751	0.634
TG 4:0_12:0_20:0	1.472	2.437	5.864	1.258	3.946	2.703	1.696
TG 6:0_14:0_16:0	1.222	1.383	1.723	0.592	0.969	0.922	0.717
TG 4:0_16:0_16:0	1.783	2.114	2.854	1.097	1.797	2.166	2.009
TG 4:0_16:0_16:0	1.839	1.992	2.869	1.050	1.996	1.875	1.865
TG 6:0_14:0_16:0	1.839	1.992	2.869	1.050	1.778	2.032	1.865
TG 6:0_14:0_16:0	1.891	2.188	2.878	1.068	2.134	2.108	1.897
TG 4:0_16:0_17:1	0.976	1.154	1.477	0.744	1.127	0.670	1.445
TG 4:0_16:0_17:0	1.151	1.366	1.682	0.587	1.019	0.859	0.381
TG 4:0_16:0_18:2	1.094	0.972	1.560	0.464	1.011	0.740	0.716
TG 4:0_16:0_18:1	0.987	1.149	1.321	0.526	0.814	0.626	0.524
TG 4:0_16:0_18:1	1.644	1.783	2.482	0.957	1.642	1.918	1.544
TG 4:0_16:0_18:0	7.264	2.110	11.706	0.962	6.538	3.198	1.361
TG 4:0_16:0_18:0	1.652	1.623	1.733	0.688	1.056	1.109	0.685

TG 10:0_12:0_16:0	1.000	1.518	2.246	0.630	0.957	1.362	0.742
TG 4:0_16:0_18:0	1.859	2.302	2.691	1.126	1.798	1.915	1.749
TG 6:0_16:0_16:0	1.859	2.302	2.691	1.126	1.798	1.915	1.749
TG 4:0_17:0_18:2	1.641	1.729	2.174	0.826	1.097	1.064	1.154
TG 4:0_17:0_18:1	1.302	1.297	1.597	0.637	1.155	0.883	0.818
TG 6:0_16:0_17:0	1.238	1.142	1.725	0.580	1.077	0.743	0.607
TG 6:0_16:0_18:2	0.763	1.078	1.093	0.545	0.749	0.702	0.676
TG 10:0_14:0_16:1	5.502	6.423	2.436	0.623	8.582	2.094	3.500
TG 4:0_18:0_18:1	1.768	1.140	2.607	0.588	0.960	0.721	0.710
TG 6:0_16:0_18:1	1.078	0.761	1.800	0.428	0.842	0.800	0.480
TG 4:0_16:0_20:0	1.335	2.175	3.732	0.875	3.293	3.285	1.372
TG 6:0_16:0_18:0	1.381	1.643	1.800	0.668	1.340	1.379	0.716
TG 6:0_17:1_18:1	1.629	1.686	2.287	0.728	1.614	1.159	0.919
TG 6:0_17:0_18:1	1.200	1.495	2.061	0.644	1.171	1.250	0.788
TG 6:0_17:0_18:1	1.101	1.901	2.588	0.749	1.580	1.486	1.051
TG 4:0_20:0_18:2	2.577	2.505	6.034	0.743	4.362	3.766	1.799
TG 16:1_6:0_20:1	1.514	1.657	2.731	0.822	1.674	1.164	0.888
TG 4:0_20:0_18:1	1.072	1.188	5.125	2.014	7.330	1.563	0.674
TG 8:0_16:0_18:1	1.705	2.210	3.003	0.796	1.246	1.381	1.158
TG 6:0_16:0_20:0	1.047	0.367	0.714	0.175	0.491	2.040	0.855
TG 12:0_14:0_16:0	1.440	1.634	2.202	0.775	1.466	1.212	0.962
TG 12:0_14:0_16:0	1.412	1.794	2.336	0.767	1.395	1.086	0.913
TG 6:0_18:1_19:1	1.197	1.399	1.759	0.614	1.223	1.096	0.944
TG 8:0_17:1_18:1	1.050	1.284	1.634	0.627	1.138	0.885	0.838
TG 10:0_16:0_17:0	1.192	0.814	1.937	0.693	1.200	1.060	0.840
TG 10:0_16:1_18:1	1.130	1.051	1.679	0.531	1.215	1.006	0.813
TG 10:0_16:0_18:2	1.132	1.051	1.670	0.530	1.207	0.992	0.811
TG 10:0_16:0_18:1	0.643	1.125	1.594	0.489	1.051	0.786	0.700
TG 10:0_16:0_18:1	0.645	1.124	1.593	0.489	1.057	0.786	0.700
TG 14:0_14:0_16:0	0.820	1.342	1.335	0.641	1.157	0.635	0.154
TG 10:0_16:0_18:0	1.363	1.116	1.214	0.750	1.381	1.083	0.595
TG 10:0_17:1_18:1	0.899	1.439	2.353	0.612	1.321	1.021	0.993
TG 14:0_14:0_18:3	1.103	1.108	2.006	0.503	1.137	0.635	0.978
TG 14:0_14:1_18:1	1.007	1.217	1.340	0.457	1.104	0.728	0.821
TG 12:0_16:0_18:1	1.327	1.632	2.081	0.607	1.230	1.034	0.943
TG 12:0_16:0_18:1	1.322	1.631	2.070	0.607	1.229	1.034	0.939
TG 14:0_16:0_16:0	1.374	1.652	1.706	0.699	1.254	1.009	1.025

TG 14:0_16:0_17:0	1.854	2.190	2.509	0.950	1.486	1.448	1.395
TG 12:0_18:1_18:2	0.672	1.037	2.001	0.665	0.751	0.806	1.025
TG 16:0_14:1_18:1	1.204	1.000	1.587	0.559	1.049	0.864	0.742
TG 14:0_16:0_18:1	1.405	1.685	2.011	0.698	1.100	1.095	0.940
TG 14:0_16:0_18:1	1.304	1.571	2.011	0.676	1.052	1.094	0.939
TG 14:0_16:0_18:0	1.461	1.432	1.582	0.652	1.353	1.022	1.295
TG 14:1_18:1_18:2	1.413	1.715	1.756	0.710	1.301	0.719	0.862
TG 14:0_18:1_18:2	1.146	0.961	1.547	0.455	0.807	0.884	0.747
TG 16:0_16:1_18:1	1.265	1.347	1.730	0.624	0.968	0.800	0.812
TG 16:0_16:0_18:1	1.326	1.552	1.554	0.576	1.168	0.897	0.850
TG 16:0_16:0_18:1	1.291	1.466	1.201	0.580	1.288	1.030	0.996
TG 16:0_16:0_18:0	1.633	2.108	2.125	0.897	1.390	1.516	1.775
TG 16:0_17:1_18:1	1.640	1.523	1.709	0.706	1.215	1.052	0.846
TG 16:0_17:0_18:1	1.328	1.471	1.709	0.686	1.213	1.239	0.823
TG 16:0_18:2_18:3	1.135	1.268	1.274	0.404	1.009	0.760	0.763
TG 16:1_18:1_18:2	0.956	1.109	1.319	0.434	0.961	0.398	0.579
TG 16:0_18:1_18:2	1.064	1.085	1.313	0.423	0.849	0.720	0.526
TG 16:0_18:1_18:1	1.160	1.224	1.476	0.542	0.875	0.830	0.817
TG 16:0_18:1_18:1	1.043	1.197	1.578	0.549	1.112	0.836	0.577
TG 16:0_18:1_18:1	1.043	1.197	1.578	0.549	1.112	0.836	0.577
TG 16:0_18:0_18:1	1.899	1.841	1.862	0.845	1.419	1.122	1.176
TG 16:0_18:0_18:0	1.128	1.401	1.284	0.593	0.937	0.660	0.863
TG 18:0_17:1_18:1	1.257	1.268	1.809	0.600	0.861	1.030	0.746
TG 17:0_18:0_18:1	1.488	1.536	1.678	0.612	0.992	0.864	0.767
TG 17:0_17:0_19:0	1.945	1.280	1.672	0.658	1.386	1.167	1.284
TG 18:1_18:2_18:3	0.983	1.230	1.446	0.601	0.894	0.841	0.679
TG 18:0_18:2_18:3	1.125	1.236	1.275	0.485	0.921	0.559	0.824
TG 18:1_18:1_18:2	1.038	1.284	0.899	0.493	0.948	0.678	0.782
TG 18:0_18:1_18:2	1.130	1.303	1.598	0.536	1.022	0.873	0.763
TG 18:0_18:1_18:1	0.930	1.205	1.587	0.413	1.030	0.855	0.606
TG 18:0_18:0_18:1	1.090	1.266	1.391	0.694	1.075	1.076	1.172
TG 16:0_18:0_20:0	1.494	1.959	1.426	0.584	1.196	0.980	0.686
TG 18:1_18:1_19:1	1.274	1.548	1.333	0.468	0.971	0.912	0.502
TG 18:0_18:1_19:1	1.078	1.787	2.001	0.568	1.198	1.224	1.182
TG 16:0_21:0_18:1	1.625	1.653	2.056	0.546	1.271	1.313	1.146
TG 16:0_16:0_23:0	1.555	1.605	2.091	0.789	1.099	1.269	1.214
TG 16:0_18:1_22:5	0.994	1.423	1.646	0.653	0.824	0.698	0.575
TG 18:1_18:1_20:1	1.324	1.086	1.637	0.592	0.751	0.871	0.882

TG 18:0_18:1_20:1	1.238	1.252	1.781	0.601	1.124	1.022	0.776
TG 16:0_22:0_18:1	1.565	1.594	1.408	0.922	1.782	1.217	1.280
TG 16:0_18:0_22:0	1.453	1.655	1.787	0.615	1.422	1.130	0.842
TG 18:0_18:2_21:2	1.450	1.546	1.665	0.675	1.237	1.036	1.270
TG 18:0_21:1_18:2	1.397	1.527	1.848	0.702	1.134	1.212	1.023
TG 16:0_18:1_23:1	1.231	0.990	1.608	0.476	0.898	0.879	0.747
TG 16:0_23:0_18:1	1.565	1.759	2.045	0.777	1.248	1.149	0.975
TG 16:0_18:0_23:0	1.184	1.592	1.715	0.652	1.092	0.932	1.082
TG 18:0_18:1_22:5	1.145	1.204	1.546	0.491	0.886	0.837	0.522
TG 18:0_18:1_22:4	0.916	0.944	1.324	0.479	0.819	0.622	0.476
TG 22:0_18:1_18:2	0.981	1.249	1.467	0.609	0.913	0.779	0.784
TG 16:0_18:1_24:1	1.004	1.411	1.764	0.642	0.922	1.013	0.798
TG 16:0_24:0_18:1	1.189	1.435	1.705	0.605	1.150	0.970	0.795
TG 16:0_18:0_24:0	0.824	1.328	1.736	0.660	1.168	0.972	0.857
TG 18:1_18:1_23:1	1.197	1.379	1.828	0.636	0.671	0.986	0.773
TG 18:0_18:1_23:1	1.245	1.431	1.853	0.623	0.997	0.763	0.850
TG 16:0_25:0_18:1	1.202	1.551	1.774	0.667	1.194	0.977	0.860
TG 16:0_18:0_25:0	1.265	1.520	1.752	0.675	1.179	1.056	0.945
TG 24:0_18:1_18:2	1.761	1.933	2.496	0.781	1.531	1.225	1.116
TG 16:0_26:0_18:2	1.304	1.886	2.506	0.801	1.541	1.332	1.086
TG 16:0_26:0_18:1	1.180	1.534	1.852	0.610	1.039	1.000	0.883
TG 16:0_18:0_26:0	1.177	1.497	1.662	0.627	1.079	1.031	0.874
TG 18:0_18:1_25:1	1.195	1.450	1.801	0.610	1.076	0.959	0.839
TG 18:0_26:0_17:1	1.237	1.471	1.803	0.634	1.215	1.048	0.828
TG 26:0_18:1_18:1	1.398	1.732	2.211	0.809	1.459	1.244	1.016
TG 18:0_26:0_18:1	1.217	1.523	1.891	0.653	1.178	1.010	0.859
TG 18:0_26:0_18:1	1.279	1.406	1.949	0.647	1.237	1.060	0.902

Table S7: Substrate specificity factors depending on the lipase (A, E, G, J, K, M or O) in pound cake (given as mean, n = 3).

	A	E	G	J	K	M	O
BisMePA 34:3	0.968	1.350	0.413	0.510	1.193	0.556	1.431
BisMePA 34:2	0.410	0.993	0.164	0.298	1.066	0.429	1.634
BisMePA 36:4	1.869	2.963	1.023	1.511	2.142	0.977	2.681
BisMePA 36:3	0.363	0.931	0.161	0.273	0.920	0.393	0.993
BisMePA 36:2	0.296	1.294	0.084	0.236	0.721	0.438	1.328
BisMePA 36:2	0.222	1.237	0.130	0.214	1.170	0.539	1.903
BisMePA 38:7	0.284	1.230	0.063	0.153	0.907	0.426	1.461
BisMePA 40:7	0.194	1.553	0.034	0.125	0.875	0.425	1.547
DG 22:4_18:3; P	1.475	1.370	0.848	1.022	1.315	0.737	1.773
DG 24:4_18:3; P	1.280	1.864	0.667	0.928	0.997	0.685	1.344
DG 26:4_18:3; P	1.346	1.694	0.555	0.919	0.929	0.527	1.225
DG 30:4_18:2; P	1.757	2.474	0.679	1.151	1.176	0.710	1.414
DG 30:4_20:4; P	1.491	1.930	0.743	0.976	1.005	0.668	1.402
DG 50:7; P	1.222	1.667	0.616	0.773	0.939	0.481	1.302
DG 30:6 ; O	1.766	3.526	1.273	1.963	2.286	0.873	2.771
DG 26:8_16:2; O	1.108	1.010	0.296	0.333	0.284	0.188	0.436
DG 42:6; O	0.463	0.856	0.211	0.849	0.211	0.312	1.133
DG 24:7_21:3; O	1.186	1.145	0.288	0.325	0.315	0.175	0.404
DG 28:6_19:0; O	1.311	1.589	0.567	0.854	0.905	0.489	1.291
DG 6:0_12:1	1.193	2.126	0.857	1.353	2.019	0.683	1.713
DG 18:1_18:3	0.910	1.169	0.466	0.633	0.771	0.403	1.173
DG 18:2_18:2	1.220	1.505	0.542	0.419	0.819	0.268	1.158
DG 18:1_18:2	2.003	1.517	1.407	1.571	1.253	0.563	1.832
DG 16:0_24:6	0.263	0.796	0.473	0.445	0.404	0.345	0.532
DG 24:6_18:0	1.368	0.716	1.018	0.811	0.885	0.347	0.832
DGDG 16:0_18:2	0.805	1.407	0.443	0.488	0.866	0.361	1.461
DGDG 18:2_18:2	1.184	1.724	0.423	0.789	0.914	0.422	1.424
DGMG 18:2	5.037	4.368	2.559	3.520	2.083	1.325	1.569
LPC 14:0	1.136	1.936	0.463	0.548	0.760	0.426	1.165
LPC 16:1	7.503	3.592	4.929	5.242	1.386	0.996	1.283
LPC 16:0	1.703	1.988	0.886	1.155	1.153	0.613	1.431
LPC 17:1	7.199	5.593	5.332	5.932	2.218	1.233	1.862
LPC 18:3	1.743	1.848	0.756	0.793	0.994	0.498	1.134
LPC 18:2	2.850	2.322	1.641	1.898	1.263	0.583	1.313
LPC 18:1	13.263	4.510	8.206	7.925	1.548	1.155	1.098

LPC 18:0	1.479	1.661	0.889	0.973	1.223	0.536	1.541
LPC 19:1	9.450	6.873	4.880	4.356	2.229	1.742	1.302
LPC 20:4	61.569	18.371	37.500	37.073	3.715	4.524	0.691
LPC 20:1	1.919	1.634	1.117	1.137	0.949	0.312	1.053
LPC 22:6	330.139	107.209	226.456	219.517	17.390	24.861	2.591
LPC 22:5	108.297	20.777	42.732	42.628	6.516	7.499	0.748
LPE 16:0	1.651	1.441	0.926	1.036	0.987	0.489	1.371
LPE 18:2	5.773	3.179	3.247	3.429	1.335	0.888	1.445
LPE 18:1	20.662	7.898	12.071	11.230	2.411	2.163	1.424
LPE 18:0	1.129	1.217	0.603	0.729	0.773	0.352	1.066
LPE 20:4	89.761	32.271	59.595	56.052	6.696	8.576	0.858
LPE 22:6	70.498	34.743	53.255	51.913	5.899	7.903	1.656
LPE 22:5	49.979	11.969	21.288	22.925	4.147	5.081	1.116
MG 18:3	2.209	1.685	1.564	1.443	2.487	0.791	1.983
MG 18:2	2.078	3.086	1.085	1.397	1.342	1.067	1.961
MG 17:3; O	1.105	3.580	1.189	1.842	2.670	0.712	2.547
MGDG 18:2_18:2	0.797	0.728	0.205	0.359	0.731	0.329	1.320
MGMG 18:2	3.296	2.977	2.030	2.596	2.115	1.117	1.722
PA 22:5_18:1	0.202	0.599	0.045	0.148	1.166	0.507	1.512
PC 39:10; P	0.849	2.592	0.267	0.855	1.511	0.567	0.481
PC 34:2 ; P	1.223	1.910	0.799	0.818	0.936	0.607	1.434
PC 18:1	2.700	3.347	1.245	1.515	1.565	0.770	1.261
PC 30:0	0.953	1.510	0.479	0.713	1.042	0.513	1.273
PC 32:3	1.507	2.215	1.039	1.491	1.854	0.742	2.209
PC 32:2	0.782	1.185	0.358	0.519	0.958	0.462	0.982
PC 32:1	0.532	1.576	0.269	0.399	1.118	0.484	1.473
PC 32:0	0.719	1.500	0.427	0.541	1.053	0.464	1.551
PC 15:2_18:2	0.773	1.447	0.488	0.794	0.745	0.452	1.131
PC 33:2	0.620	1.515	0.380	0.508	0.978	0.474	1.189
PC 33:1	0.423	1.098	0.105	0.236	0.829	0.447	1.369
PC 34:4	0.582	1.572	0.321	0.471	0.919	0.467	1.246
PC 34:3	0.850	1.605	0.397	0.615	0.945	0.472	1.239
PC 16:0_18:2	0.557	1.232	0.273	0.337	0.836	0.393	1.240
PC 34:2	1.979	5.693	0.899	1.253	2.186	0.347	3.580
PC 34:1	0.375	1.308	0.166	0.269	0.649	0.328	1.387
PC 16:0_18:1	0.376	1.228	0.177	0.266	0.993	0.455	1.429
PC 35:2	0.420	1.417	0.227	0.352	0.939	0.478	1.400

PC 35:1	0.192	1.049	0.058	0.153	0.780	0.390	1.289
PC 36:6	2.457	4.047	1.164	1.629	2.115	1.017	2.449
PC 36:5	1.182	2.096	0.581	0.853	1.110	0.552	1.239
PC 36:5	1.478	1.873	0.728	0.953	1.135	0.227	1.005
PC 18:2_18:2	0.885	1.485	0.412	0.637	0.929	0.462	1.165
PC 18:2_18:2	0.947	1.592	0.446	0.632	0.942	0.455	1.201
PC 36:4	0.853	1.523	0.422	0.617	0.883	0.415	1.105
PC 18:1_18:2	0.957	1.835	0.413	0.687	0.808	0.369	1.051
PC 36:2	0.438	1.377	0.205	0.326	0.943	0.467	1.340
PC 18:0_18:2	0.392	1.373	0.199	0.273	0.931	0.455	1.398
PC 18:0_18:1	0.568	1.480	0.224	0.436	1.092	0.610	1.585
PC 36:1	0.448	1.303	0.209	0.330	0.835	0.501	1.787
PC 38:7	0.764	0.995	0.184	0.176	0.342	0.303	0.606
PC 16:0_22:6	0.497	2.010	0.234	0.429	1.265	0.528	1.622
PC 18:0_20:4	0.467	1.480	0.188	0.354	1.022	0.568	1.466
PC 40:9	0.195	1.498	0.053	0.092	0.685	0.330	0.960
PC 40:7	0.065	0.677	0.041	0.016	0.138	0.130	0.559
PC 40:6	0.477	1.887	0.263	0.432	1.116	0.547	1.515
PC 40:5	0.398	1.067	0.252	0.496	1.322	0.595	1.737
PE P-18:0	21.749	7.806	12.678	11.684	2.487	2.279	1.459
PE 16:0_18:2	0.353	1.081	0.118	0.233	0.908	0.402	1.255
PE 16:0_18:1	0.247	1.138	0.088	0.141	0.910	0.405	1.385
PE 18:2_18:2	0.835	1.507	0.554	0.840	0.901	0.534	1.230
PE 18:2_18:2	1.642	1.844	0.669	0.934	0.955	0.451	1.181
PE 20:4_16:0	0.614	1.008	0.374	0.549	0.899	0.299	1.080
PE 36:3	1.310	2.352	0.688	0.988	1.585	0.653	1.976
PE 18:0_18:2	0.258	1.163	0.089	0.181	0.864	0.394	1.398
PE 18:0_18:1	0.108	1.177	0.092	0.167	0.732	0.280	1.537
PE 16:0_22:6	0.269	1.382	0.064	0.177	0.851	0.420	1.305
PE 20:4_18:1	0.107	0.496	0.024	0.102	0.815	0.365	1.234
PE 18:0_20:4	0.531	1.444	0.226	0.368	0.970	0.444	1.519
PEt 10:0_12:1	1.249	1.648	0.668	0.740	0.892	0.567	1.257
PEt 10:0_18:2	1.099	1.558	0.616	0.689	1.001	0.571	1.245
PEt 10:0_18:1	1.186	1.584	0.561	0.727	0.898	0.527	1.263
PEt 10:0_20:2	1.142	1.583	0.579	0.683	0.940	0.532	1.181
PEt 10:0_22:1	1.063	1.974	0.737	0.815	1.757	0.542	1.046
PEt 10:0_22:1	1.190	2.010	0.586	0.731	1.401	0.401	0.591
PEt 10:0_24:1	1.235	1.937	0.617	0.850	1.159	0.568	1.863

PEt 10:0_24:1	1.177	2.313	0.630	0.980	1.264	0.561	1.731
PIP3 P-6:0_16:3	2.596	3.951	1.679	2.398	2.448	1.270	3.140
PIP3 O-4:0_18:4	3.602	4.425	1.603	2.479	2.785	1.385	3.154
SM d32:1	1.185	1.685	0.624	0.742	1.031	0.598	1.405
SM d34:1	0.933	0.989	0.608	0.660	0.558	0.424	1.031
SM d34:0	0.833	2.251	0.625	0.791	0.985	0.524	1.493
SM d36:1	1.294	1.685	0.743	0.865	1.051	0.579	1.398
SM d40:1	0.626	0.522	0.434	0.574	0.584	0.364	0.605
SM d42:1	1.148	1.490	0.428	0.426	0.534	0.425	0.449
SPB d14:0	1.825	2.488	1.094	1.578	1.650	1.095	2.676
SPB d18:0	1.452	0.966	0.975	1.856	1.281	0.874	2.060
SPB d14:1	1.273	2.422	1.046	1.355	1.560	0.712	1.628
SPB d16:1	0.982	1.958	0.938	1.466	1.336	0.806	2.009
SPB d18:1	4.816	6.248	2.050	3.432	3.650	1.931	4.348
TG 25:6_27:8; P	2.097	2.680	0.741	1.185	2.657	1.378	3.487
TG 4:0_6:0_10:0	1.256	1.797	0.894	1.142	1.981	0.916	3.531
TG 4:0_4:0_14:1	2.231	3.401	1.020	1.544	1.916	0.919	2.091
TG 4:0_6:0_12:0	1.595	2.218	0.699	1.148	1.359	0.702	1.702
TG 4:0_6:0_14:1	1.058	1.641	0.539	0.651	0.831	0.502	1.279
TG 8:0_8:0_8:0	2.328	3.205	1.063	1.620	2.221	1.047	2.418
TG 2:0_10:0_12:0	1.260	1.782	0.683	0.841	1.100	0.610	1.406
TG 4:0_4:0_18:2	1.376	2.118	0.663	1.073	1.415	0.725	1.149
TG 4:0_4:0_18:1	1.128	1.784	0.465	0.779	1.013	0.503	1.273
TG 8:0_8:0_10:0	2.271	2.703	1.038	1.490	1.762	1.228	3.118
TG 4:0_6:0_16:0	1.296	1.722	0.663	0.799	1.054	0.593	1.453
TG 4:0_6:0_17:1	1.427	1.691	0.681	0.726	1.151	0.582	1.485
TG 4:0_6:0_18:2	1.271	1.708	0.659	0.831	1.114	0.612	1.345
TG 4:0_6:0_18:1	1.451	1.297	0.728	0.747	1.171	0.762	1.868
TG 8:0_10:0_10:0	1.105	19.467	4.840	13.035	16.472	7.328	12.270
TG 4:0_10:0_14:0	1.377	1.735	0.706	0.874	0.874	0.321	1.502
TG 4:0_8:0_17:1	1.301	1.678	0.708	0.849	1.237	0.641	1.471
TG 4:0_8:0_18:2	1.480	2.599	0.906	1.082	1.388	0.842	2.320
TG 4:0_8:0_18:1	1.276	1.647	0.650	0.782	1.052	0.581	1.418
TG 4:0_6:0_20:0	1.051	5.554	0.550	0.810	1.071	0.808	2.497
TG 4:0_10:0_16:0	1.325	1.744	0.676	0.738	1.019	0.607	1.318
TG 4:0_10:0_16:0	1.274	2.218	0.597	0.669	0.829	0.486	1.315
TG 6:0_8:0_17:1	1.436	2.410	0.782	1.048	1.610	0.391	1.551

TG 6:0_8:0_18:2	1.329	1.438	0.655	0.778	0.898	0.508	1.260
TG 4:0_14:0_14:1	1.101	1.616	0.639	0.717	0.923	0.527	1.300
TG 4:0_8:0_20:0	0.924	1.324	6.043	0.724	1.010	0.723	2.410
TG 4:0_12:0_16:0	1.415	1.638	0.736	0.731	1.366	0.632	1.780
TG 4:0_12:0_16:0	1.192	1.592	0.749	0.882	1.161	0.628	1.934
TG 4:0_15:0_14:1	1.173	1.338	0.568	0.659	0.993	0.459	1.159
TG 6:0_10:0_17:1	1.236	1.772	1.124	0.916	1.270	0.630	1.285
TG 4:0_12:0_18:2	1.471	1.743	0.732	0.546	0.927	0.623	1.349
TG 4:0_12:1_18:1	1.481	1.717	0.682	0.716	0.933	0.627	1.359
TG 4:0_12:0_18:1	1.243	1.407	0.586	0.696	0.800	0.483	1.221
TG 4:0_12:0_18:1	1.366	1.609	0.511	0.872	0.958	0.325	1.387
TG 4:0_10:0_20:0	0.974	1.262	0.507	0.760	1.035	0.665	2.020
TG 4:0_14:0_16:0	1.317	1.516	0.795	0.830	1.030	0.508	1.704
TG 4:0_14:0_16:0	1.370	1.323	0.548	0.711	0.654	0.540	0.903
TG 8:0_10:0_17:1	1.107	1.420	0.538	0.644	0.896	0.415	1.455
TG 8:0_10:0_17:1	1.161	1.420	0.559	0.582	0.808	0.415	1.455
TG 4:0_14:0_18:3	0.700	0.829	0.547	0.518	0.867	0.474	1.022
TG 4:0_14:0_18:3	1.068	1.040	0.484	0.507	0.813	0.489	1.169
TG 4:0_14:0_18:2	1.108	1.633	0.655	0.881	0.668	0.548	2.126
TG 4:0_14:0_18:1	1.097	1.481	0.534	0.784	1.042	0.668	2.316
TG 4:0_14:0_18:1	1.134	1.904	0.603	0.693	1.046	0.489	1.294
TG 4:0_14:0_18:1	1.590	1.952	0.935	0.980	1.046	0.919	1.294
TG 4:0_14:0_18:1	1.164	1.645	0.478	0.795	1.091	0.472	1.162
TG 4:0_16:0_16:0	1.259	1.603	0.628	0.797	1.092	0.643	2.179
TG 6:0_14:0_16:0	1.222	1.485	0.697	0.656	1.015	0.543	1.373
TG 4:0_16:0_16:0	1.681	1.890	0.919	0.856	0.779	0.579	1.428
TG 4:0_16:0_16:0	1.008	1.826	0.782	0.815	1.018	0.569	1.401
TG 4:0_16:0_17:0	1.370	1.855	0.709	0.616	1.284	0.513	1.247
TG 6:0_14:0_17:0	1.259	1.795	0.691	0.532	1.097	0.504	1.163
TG 6:0_14:0_17:0	1.005	1.715	0.661	0.502	1.081	0.581	0.909
TG 30:2_4:0_4:0	1.372	1.533	0.432	0.732	1.131	0.723	2.091
TG 4:0_16:1_18:1	0.920	1.220	0.546	0.509	0.648	0.372	1.078
TG 4:0_16:0_18:2	0.995	1.473	0.644	0.813	0.753	0.579	1.269
TG 4:0_16:0_18:1	1.132	1.416	0.564	0.813	1.070	0.561	2.041
TG 4:0_16:0_18:1	1.187	1.611	0.566	0.708	0.912	0.724	1.607
TG 4:0_16:0_18:1	1.226	1.610	0.670	0.799	1.052	0.616	1.092
TG 4:0_16:0_18:0	1.088	1.235	0.406	0.669	0.874	0.640	1.730
TG 4:0_16:0_18:0	1.338	1.779	0.642	0.712	1.060	0.677	1.810

TG 10:0_12:0_16:0	1.434	1.889	0.713	0.786	1.026	0.534	1.572
TG 4:0_16:0_18:0	1.686	2.150	0.465	0.909	1.004	0.816	1.485
TG 4:0_17:1_18:1	1.109	1.967	0.722	0.793	0.626	0.618	1.307
TG 4:0_17:0_18:1	0.956	1.784	0.854	0.485	1.147	0.503	1.252
TG 4:0_17:0_18:1	0.700	1.495	0.674	0.595	0.940	0.380	0.963
TG 4:0_18:1_18:2	2.565	1.670	1.070	1.383	1.606	0.971	2.499
TG 4:0_18:1_18:1	0.835	1.037	0.439	0.575	0.797	0.554	1.183
TG 4:0_18:1_18:1	1.261	1.684	0.683	0.844	1.060	0.573	1.471
TG 10:0_12:0_18:2	1.261	1.684	0.755	0.844	1.060	0.573	1.471
TG 4:0_18:0_18:1	1.270	1.154	0.504	0.847	1.043	0.409	2.067
TG 4:0_18:0_18:1	1.331	1.421	0.488	0.640	0.841	0.511	1.377
TG 6:0_16:0_18:1	1.194	1.696	0.641	0.764	1.045	0.529	1.107
TG 4:0_16:0_20:0	0.963	1.496	0.514	0.701	0.875	0.609	1.659
TG 6:0_16:0_18:0	0.810	1.024	0.342	0.746	0.659	0.370	1.028
TG 10:0_14:0_16:0	1.179	1.384	0.547	0.604	0.841	0.525	1.223
TG 4:0_18:1_19:1	1.782	2.084	0.688	0.868	0.963	0.642	1.765
TG 6:0_17:1_18:1	4.620	1.325	2.474	2.452	1.830	1.438	3.711
TG 6:0_17:1_18:1	1.876	2.281	0.732	0.934	0.834	0.613	2.009
TG 18:0_4:0_19:1	0.738	1.554	0.437	0.792	0.882	0.468	1.119
TG 4:0_20:0_18:2	0.996	0.935	0.367	0.526	0.891	0.522	0.965
TG 4:0_20:0_18:2	3.407	4.049	0.899	2.431	3.712	2.345	2.272
TG 6:0_18:1_18:1	0.469	0.601	0.256	0.436	0.403	0.216	0.615
TG 6:0_18:1_18:1	1.468	1.917	0.750	0.888	1.227	0.685	1.698
TG 4:0_20:0_18:1	1.569	2.457	0.696	0.913	1.545	0.508	1.819
TG 10:0_14:0_18:1	1.544	1.578	1.142	0.894	1.264	0.804	1.410
TG 8:0_16:0_18:1	1.554	1.632	1.165	0.924	1.282	0.804	1.424
TG 8:0_16:0_18:1	1.275	1.711	0.599	0.839	0.933	0.540	1.275
TG 6:0_16:0_20:0	1.075	1.222	0.563	0.672	0.915	0.491	1.465
TG 8:0_16:0_18:0	0.733	1.093	0.358	0.451	1.249	0.660	0.949
TG 12:0_14:0_16:0	1.126	1.529	0.562	0.603	0.850	0.488	1.038
TG 8:0_17:1_18:1	1.491	2.161	0.818	0.960	1.018	0.611	1.752
TG 10:0_16:0_17:1	0.576	0.608	0.712	0.303	0.401	0.258	1.336
TG 10:0_16:0_18:2	0.743	1.400	0.360	0.995	1.277	0.301	1.061
TG 10:0_16:0_18:2	0.489	1.583	0.168	0.317	0.483	0.264	1.742
TG 10:0_16:0_18:1	1.659	1.411	0.568	0.625	0.579	0.537	1.519
TG 10:0_16:0_18:1	1.185	1.648	0.598	0.733	0.910	0.496	1.254
TG 8:0_16:0_20:0	1.428	2.317	0.879	0.632	0.432	0.279	1.124

TG 10:0_16:0_18:0	1.255	1.635	0.627	0.734	0.744	0.566	1.309
TG 8:0_18:1_19:1	1.139	1.667	0.651	0.552	1.163	0.302	0.331
TG 8:0_18:1_19:1	0.740	2.015	0.771	0.697	1.161	0.332	1.603
TG 12:0_17:0_16:1	1.077	1.540	0.636	0.721	0.971	0.434	1.168
TG 10:0_18:0_18:3	1.036	1.520	0.787	1.084	1.429	0.831	1.546
TG 14:0_14:0_18:3	0.781	1.769	0.679	0.567	1.236	0.364	0.977
TG 14:0_14:1_18:1	1.176	1.477	0.578	0.663	0.862	0.506	1.288
TG 14:0_14:1_18:1	1.146	1.501	0.586	0.608	0.897	0.512	1.243
TG 12:0_16:0_18:1	1.415	1.615	0.961	1.108	1.917	0.685	2.004
TG 14:0_16:0_16:0	1.146	1.300	0.474	0.659	0.869	0.475	1.186
TG 14:0_14:1_19:1	1.126	1.638	0.708	0.815	1.103	0.512	1.531
TG 14:0_16:0_17:0	0.621	1.451	0.453	0.556	0.763	0.353	1.150
TG 14:1_16:1_18:2	1.288	1.481	0.650	0.663	0.789	0.583	1.466
TG 12:0_18:1_18:2	1.081	1.971	0.560	0.691	1.153	0.625	1.469
TG 12:0_18:1_18:2	1.083	1.972	0.562	0.691	1.158	0.626	1.470
TG 12:0_18:1_18:1	1.002	1.887	0.341	0.845	1.089	0.501	0.819
TG 16:0_14:1_18:1	0.959	1.466	0.263	0.727	0.933	0.078	0.285
TG 14:0_16:0_18:1	1.444	1.076	0.958	0.911	1.781	0.635	1.864
TG 14:0_16:0_18:0	1.263	1.907	0.719	0.867	0.652	0.316	0.954
TG 14:1_18:1_18:2	0.673	1.860	0.494	0.731	0.938	0.394	1.288
TG 14:0_18:1_18:3	0.608	1.864	0.337	0.738	0.890	0.395	1.036
TG 14:0_18:1_18:2	1.230	1.385	1.039	1.602	1.511	0.964	2.239
TG 16:0_16:1_18:1	1.318	1.594	0.600	0.757	0.943	0.505	0.728
TG 16:0_16:0_18:2	6.600	9.355	3.327	4.132	5.252	2.512	5.994
TG 16:0_16:0_18:1	1.075	1.498	0.554	0.740	0.920	0.521	1.332
TG 16:0_16:0_18:0	1.104	1.457	0.466	0.477	0.539	0.327	0.874
TG 16:0_17:1_18:1	0.775	1.383	0.596	0.625	0.792	0.520	1.237
TG 16:0_17:1_18:1	0.863	1.451	0.620	0.728	0.859	0.388	0.921
TG 16:0_17:0_18:1	2.019	1.512	1.042	1.290	1.490	0.600	2.105
TG 16:0_17:0_18:1	1.704	2.841	1.227	1.276	1.053	0.809	2.196
TG 17:0_17:0_17:0	1.209	1.688	0.646	0.834	0.863	0.424	1.148
TG 16:0_17:0_18:0	1.155	1.984	0.675	0.939	1.155	0.432	1.203
TG 16:0_18:2_18:3	1.423	2.178	0.812	0.973	1.159	0.565	1.599
TG 16:1_18:1_18:2	1.085	1.173	0.579	0.671	0.971	0.288	1.400
TG 16:1_18:1_18:2	1.083	0.959	0.578	0.670	0.969	0.319	1.380
TG 16:0_18:2_18:2	12.673	14.715	5.009	7.880	8.062	3.213	6.112
TG 16:0_18:2_18:2	1.451	2.220	0.785	1.352	1.431	0.683	1.555
TG 16:0_18:1_18:2	1.791	1.737	0.881	0.707	1.128	0.431	1.276

TG 16:0_18:1_18:1	1.158	1.530	0.512	0.598	0.434	0.417	1.187
TG 16:0_18:1_18:1	5.605	6.818	2.615	3.438	3.947	2.077	4.986
TG 16:0_18:1_18:1	0.992	1.562	0.557	0.676	0.561	0.458	1.219
TG 16:0_18:1_18:1	1.058	1.634	0.579	0.670	0.570	0.488	1.195
TG 16:0_18:0_18:1	1.737	1.951	0.824	0.575	0.815	0.460	1.175
TG 16:0_18:0_18:0	1.367	1.739	0.626	0.572	0.915	0.538	1.001
TG 17:0_18:1_18:1	1.166	1.339	0.631	0.686	0.962	0.396	1.080
TG 18:0_17:1_18:1	1.055	1.478	0.343	0.716	0.945	0.353	0.775
TG 17:0_18:0_18:1	0.783	1.386	0.507	0.549	0.639	0.435	0.845
TG 18:2_18:2_18:3	2.703	3.464	1.138	1.655	1.768	0.937	1.819
TG 17:0_17:0_19:0	1.063	1.544	0.497	0.574	0.572	0.357	0.907
TG 18:1_18:2_18:3	2.060	2.017	0.460	1.214	1.669	0.818	1.608
TG 18:1_18:2_18:2	1.559	2.564	0.883	0.980	1.448	0.666	1.302
TG 18:1_18:1_18:2	1.130	1.863	0.655	0.774	1.077	0.491	1.262
TG 18:1_18:1_18:2	1.332	1.443	0.614	0.530	0.857	0.407	0.994
TG 18:0_18:2_18:2	8.438	11.066	3.886	5.505	6.303	2.962	7.261
TG 18:0_18:1_18:2	1.246	1.727	0.686	0.793	0.840	0.532	1.355
TG 18:0_18:1_18:2	1.048	1.614	0.686	0.656	0.840	0.370	0.909
TG 18:0_18:1_18:1	1.346	2.087	0.776	0.748	1.161	0.596	1.479
TG 18:0_18:0_18:1	0.982	1.338	0.367	0.345	0.557	0.366	0.771
TG 16:0_18:0_20:0	0.648	1.297	0.310	0.268	0.294	0.225	0.558
TG 18:1_18:1_19:1	1.002	1.957	0.562	0.881	1.006	0.519	1.391
TG 18:0_18:1_19:1	0.966	1.292	0.613	0.340	0.743	0.433	1.104
TG 16:0_21:0_18:1	1.030	1.208	0.532	0.509	0.667	0.353	0.630
TG 16:0_16:0_23:0	1.346	2.034	0.683	0.826	0.877	0.479	1.489
TG 16:0_18:1_22:5	1.367	1.870	0.716	0.869	1.057	0.568	1.489
TG 18:1_18:1_20:1	1.098	1.901	0.576	0.842	0.846	0.471	1.335
TG 18:0_18:1_20:1	1.511	2.129	0.734	0.783	1.167	0.308	1.454
TG 16:0_22:0_18:1	1.016	1.332	0.515	0.698	0.879	0.518	0.922
TG 16:0_18:0_22:0	1.233	1.883	0.719	0.700	0.993	0.596	1.386
TG 16:1_18:1_23:1	1.087	1.540	0.589	0.719	0.841	0.472	1.157
TG 16:0_18:1_23:1	1.326	2.430	0.506	1.054	1.169	0.754	1.391
TG 16:0_23:0_18:1	1.216	1.522	0.581	0.642	0.946	0.487	1.112
TG 16:0_18:0_23:0	1.347	1.794	0.624	0.806	1.020	0.560	1.280
TG 18:0_18:1_22:5	1.157	1.387	0.590	0.647	0.725	0.410	1.164
TG 18:0_18:1_22:4	1.370	1.266	0.765	0.921	1.137	0.576	1.453
TG 22:0_18:1_18:3	1.713	2.387	0.827	1.104	1.349	0.691	1.594

TG 22:0_18:1_18:2	1.480	2.212	0.557	1.108	1.337	0.711	1.732
TG 16:0_18:1_24:1	1.234	1.972	0.647	0.965	0.929	0.558	1.655
TG 16:0_24:0_18:1	1.113	1.345	0.644	0.700	0.815	0.427	1.117
TG 16:0_18:0_24:0	1.305	1.745	0.645	0.726	0.964	0.529	1.293
TG 18:1_18:1_23:1	2.638	3.819	1.415	1.836	1.923	1.091	2.632
TG 18:0_18:1_23:1	1.356	1.673	0.520	0.741	1.038	0.540	1.243
TG 16:0_25:0_18:1	1.207	1.833	0.773	0.934	1.152	0.593	1.404
TG 16:0_18:0_25:0	1.500	2.090	0.779	0.893	1.169	0.523	1.465
TG 24:0_18:1_18:2	1.866	2.562	0.807	1.091	1.330	0.652	1.815
TG 16:0_26:0_18:2	1.203	1.931	0.502	0.995	0.975	0.539	1.339
TG 16:0_26:0_18:1	1.271	1.715	0.628	0.648	1.037	0.466	0.851
TG 16:0_18:0_26:0	1.617	2.136	0.676	0.883	1.282	0.541	1.459
TG 18:0_26:0_17:1	1.566	1.837	0.773	0.904	1.087	0.636	1.518
TG 26:0_18:1_18:1	1.635	2.114	0.825	1.019	1.184	0.613	1.573
TG 18:0_26:0_18:1	1.612	2.331	0.855	1.015	1.227	0.662	1.655

Table S8: Substrate specificity factors depending on the lipase (A, E, G, J, K, M or O) in brioche dough (given as mean, n = 3).

	A	E	G	J	K	M	O
BisMePA 34:3	0.791	1.337	1.388	0.528	1.679	1.162	0.717
BisMePA 34:2	1.858	1.333	1.543	0.553	2.141	1.190	1.468
BisMePA 36:5	0.829	1.268	1.543	0.515	4.208	1.259	0.771
BisMePA 36:3	1.731	1.579	1.936	0.684	1.326	1.300	1.175
DG 24:1; P	0.784	1.324	1.495	0.761	2.138	0.995	0.660
DG 46:5; P	0.730	0.974	0.707	0.578	0.551	1.072	0.335
DG 25:8_23:6; P	1.158	1.484	1.764	0.701	1.100	1.216	0.797
DG 30:4_18:2; P	0.809	1.717	1.521	0.846	2.436	1.058	0.943
DG 30:6; O	0.692	1.225	1.121	0.667	1.301	1.208	1.036
DG 30:6_19:0; O	0.728	1.342	1.187	0.714	1.165	0.856	0.742
DG 6:0_12:1	1.629	1.548	2.131	0.509	1.587	2.359	1.711
DGDG 16:0_18:2	0.625	1.209	1.539	0.529	1.831	1.149	0.851
DGDG 18:2_18:3	1.389	1.414	1.621	0.524	1.591	1.180	0.906
DGDG 18:2_18:2	1.509	1.873	2.137	0.863	2.057	1.590	1.455
DGMG 18:2	0.485	0.687	1.241	0.424	1.782	0.866	0.443
LPC 16:1	0.999	0.960	1.536	0.371	2.468	1.245	0.871
LPC 18:1	0.524	0.793	1.350	0.435	1.051	1.095	0.458
LPC 18:0	1.086	1.021	1.393	0.374	1.594	1.071	1.048
LPC 20:4	0.158	0.715	1.385	0.610	1.433	1.671	0.221
LPC 22:6	0.039	0.359	0.950	0.315	1.668	0.997	0.098
LPC 22:5	0.026	0.306	0.959	0.321	1.601	1.001	0.089
LPC 12:1; O	0.647	0.788	1.429	0.652	1.130	1.044	0.334
LPE 16:0	1.088	0.825	1.174	0.392	0.942	1.052	0.721
LPE 18:2	0.786	1.274	1.386	0.560	0.528	2.209	0.724
LPE 18:2	0.681	0.707	1.109	0.369	1.574	1.048	0.451
LPE 18:1	0.216	0.502	0.969	0.298	1.711	1.077	0.241
LPE 20:4	0.016	0.434	1.015	0.283	1.153	1.351	0.082
MG 18:2	0.545	0.865	1.602	0.354	1.061	0.917	0.554
MGDG 18:2_18:2	2.010	1.606	1.962	0.764	1.400	1.189	1.153
MGMG 18:2	0.439	0.582	1.102	0.330	1.008	0.688	0.382
PC 28:5_20:5; O	1.164	1.191	1.540	0.607	1.921	1.127	0.896
PC 30:0	0.838	1.636	1.294	0.895	0.881	1.109	0.924
PC 32:2	1.146	1.440	1.869	0.564	1.624	0.673	0.720
PC 32:1	1.410	1.388	1.558	0.524	1.350	1.167	1.086

PC 32:0	1.483	1.724	1.703	0.689	1.329	1.296	1.185
PC 33:4	0.896	1.049	1.502	0.376	1.066	0.817	0.606
PC 34:3	1.203	1.137	1.480	0.506	0.987	1.055	0.797
PC 16:0_18:2	1.228	1.266	1.730	0.563	1.315	1.150	0.958
PC 16:0_18:1	1.395	0.991	1.601	0.367	1.577	1.060	1.053
PC 34:1	1.615	1.272	1.525	0.268	1.996	0.883	1.040
PC 18:2_18:2	1.087	1.307	1.633	0.563	2.173	1.158	0.867
PC 18:0_18:2	1.868	1.276	1.580	0.492	2.159	1.057	1.058
PC 36:1	1.591	1.153	1.416	0.339	1.629	0.961	1.093
PC 38:6	1.724	0.985	1.731	0.367	1.893	0.944	1.046
PC 38:4	1.628	1.214	1.331	0.329	2.314	0.770	0.979
PE 18:2_18:2	0.872	0.947	0.996	0.441	2.341	0.897	0.646
PEt 26:3_18:4; O	1.026	1.180	1.441	0.588	1.955	0.877	0.794
PEt 10:0_18:1	0.798	1.275	1.247	0.743	1.341	1.117	0.783
PEt 24:6_18:2	0.990	1.657	1.700	0.581	1.414	0.971	0.968
PIP2 16:3_6:0	1.117	1.279	1.684	0.724	1.430	1.415	0.940
PIP2 28:6	1.078	1.328	1.527	0.592	1.405	1.079	0.887
PIP3 P-6:0_16:3	1.011	17.100	2.042	0.678	1.873	2.053	0.867
SM d34:1	1.271	1.327	1.427	0.524	1.501	0.826	0.729
SPB d14:0	1.295	1.598	2.220	0.613	5.305	2.283	1.090
SPB d18:2	1.290	0.909	1.543	0.325	1.730	1.007	0.730
SPB d18:0	1.833	2.203	3.364	0.833	2.161	2.615	1.195
SPB d14:1	1.066	1.393	2.238	0.768	1.790	1.108	1.073
SPB d16:1	1.434	2.117	3.403	1.181	2.385	2.027	1.422
SPB d18:1	0.721	0.633	1.841	0.760	1.383	1.010	0.849
TG 32:8_24:6; P	0.978	1.406	2.216	0.603	2.577	1.299	0.683
TG 20:6_21:6_21:6; P	3.071	4.392	5.380	1.823	1.715	3.121	2.133
TG 38:10_25:7; P	1.087	0.928	1.707	0.933	2.464	1.081	1.182
TG 25:8_22:6_22:6; P	1.365	2.175	2.663	0.915	4.820	1.864	1.214
TG 4:0_14:0; O	1.580	1.486	2.176	0.542	3.509	1.730	1.682
TG 25:7_27:8 ; O	1.104	1.356	1.618	0.618	3.116	1.217	1.006
TG 28:8_27:7; O	0.837	0.294	1.055	0.440	3.329	1.345	0.418
TG 4:0_6:0_12:0	0.540	1.336	1.276	0.626	2.183	0.951	1.555
TG 4:0_8:0_12:0	0.534	0.829	1.301	0.509	1.748	0.832	0.760
TG 4:0_4:0_18:1	0.757	1.379	1.483	0.841	1.115	1.157	0.678
TG 4:0_6:0_16:0	0.462	1.236	1.035	0.787	0.762	0.939	0.775
TG 4:0_6:0_18:1	0.589	1.484	1.044	0.645	1.591	0.943	0.610
TG 8:0_10:0_10:0	0.903	1.308	1.072	1.088	1.330	2.073	1.053

TG 4:0_10:0_14:0	0.838	1.504	1.246	0.629	0.997	0.828	0.791
TG 4:0_8:0_18:2	0.748	1.021	1.220	0.506	2.259	1.074	0.788
TG 4:0_8:0_18:1	0.659	0.838	1.168	0.445	1.322	0.921	0.636
TG 6:0_10:0_14:0	0.784	1.117	1.004	1.220	1.351	1.836	1.093
TG 4:0_10:0_16:0	0.737	1.342	1.255	0.831	1.335	1.190	0.835
TG 4:0_8:0_18:0	0.728	1.408	1.233	0.776	2.224	1.100	0.762
TG 4:0_10:0_17:1	0.758	1.514	1.323	0.882	1.564	1.156	0.878
TG 4:0_10:0_18:2	0.933	1.687	2.124	1.012	1.382	1.725	1.307
TG 4:0_10:0_18:1	0.875	1.549	1.538	0.723	1.270	0.981	1.013
TG 6:0_8:0_18:1	0.792	1.401	1.391	0.654	1.196	1.270	0.818
TG 4:0_12:0_16:0	0.749	1.352	1.012	0.917	1.270	1.230	0.729
TG 6:0_8:0_18:0	0.602	1.567	1.483	0.537	1.270	0.797	0.695
TG 6:0_10:0_17:0	0.477	1.478	1.448	0.638	0.924	1.140	0.836
TG 4:0_12:0_18:3	0.764	1.195	1.467	0.869	0.499	0.932	0.773
TG 4:0_12:0_18:2	0.940	1.864	1.325	1.060	1.597	1.279	1.010
TG 12:0_4:0_18:1	0.869	1.313	1.271	1.346	1.325	1.885	1.196
TG 4:0_12:0_18:1	0.679	1.488	1.537	1.049	0.744	1.110	0.994
TG 6:0_10:0_18:1	0.779	0.973	1.475	0.607	2.350	0.825	0.783
TG 4:0_14:0_17:1	0.859	1.030	1.546	0.973	1.238	1.242	0.934
TG 6:0_10:0_20:3	0.714	1.299	1.342	0.818	1.134	0.979	0.622
TG 4:0_14:0_18:2	0.371	1.281	1.537	0.555	98.063	1.250	1.026
TG 4:0_14:0_18:1	0.684	1.714	1.269	1.029	1.288	1.184	0.855
TG 6:0_12:0_18:1	0.726	1.322	1.481	0.704	1.212	1.131	0.781
TG 4:0_16:0_16:0	1.266	3.343	1.166	1.586	0.896	1.373	1.447
TG 4:0_16:0_17:0	0.392	1.093	1.153	0.405	1.021	1.027	0.717
TG 4:0_16:0_18:3	0.890	1.348	1.257	0.920	1.317	1.173	0.787
TG 4:0_16:0_18:2	0.690	1.276	1.758	0.935	1.246	1.454	0.813
TG 4:0_16:1_18:1	0.962	1.985	2.104	1.259	0.452	2.113	0.841
TG 4:0_16:0_18:1	0.868	1.283	0.618	34.126	1.154	34.103	57.164
TG 6:0_14:0_18:0	0.830	1.326	1.018	1.284	0.607	1.651	1.216
TG 4:0_16:0_18:0	0.286	1.645	0.453	0.941	1.097	0.695	0.559
TG 4:0_17:1_18:1	1.029	3.422	0.997	1.609	111.725	1.981	1.221
TG 4:0_17:0_18:1	0.441	1.134	1.168	0.324	2.334	0.693	0.842
TG 4:0_18:1_18:2	0.648	1.004	1.242	0.789	1.277	0.438	0.762
TG 4:0_18:1_18:1	0.738	1.755	1.778	1.577	0.760	1.517	1.420
TG 6:0_16:0_18:1	0.212	0.892	0.333	0.278	1.461	0.778	0.237
TG 4:0_16:0_20:0	0.987	1.267	1.306	1.018	1.433	1.908	1.077

TG 10:0_14:0_16:0	0.701	0.865	0.637	0.547	1.575	1.170	0.819
TG 6:0_16:0_18:0	0.565	1.124	1.025	0.759	0.350	0.967	0.672
TG 10:0_14:0_16:0	0.740	1.328	1.252	0.709	2.449	1.116	0.805
TG 6:0_17:0_18:1	0.681	1.411	1.200	0.770	1.453	1.122	0.797
TG 6:0_18:1_18:3	0.638	1.314	1.111	0.541	1.234	0.600	0.468
TG 6:0_18:0_18:2	1.718	2.188	1.845	0.897	1.230	1.112	0.648
TG 6:0_18:1_18:1	0.816	1.367	1.421	0.453	1.398	1.271	0.841
TG 4:0_20:0_18:1	1.225	1.324	1.452	0.829	1.049	1.099	0.752
TG 12:0_14:0_16:1	0.799	0.775	1.842	0.678	0.393	1.107	0.733
TG 8:0_16:0_18:1	0.601	1.138	1.496	0.592	0.901	0.900	0.738
TG 10:0_12:0_20:0	0.947	1.031	1.235	1.161	2.997	1.509	1.294
TG 12:0_14:0_16:0	0.960	2.431	2.221	1.251	1.217	1.354	1.222
TG 8:0_17:1_18:1	0.698	1.226	1.361	0.654	1.026	1.164	0.771
TG 10:0_16:0_17:1	0.959	1.007	1.277	0.881	2.241	1.425	0.749
TG 8:0_18:1_18:3	0.717	1.420	1.261	0.612	2.544	1.198	0.757
TG 10:0_16:0_18:3	0.484	1.251	1.447	0.382	1.408	0.820	0.651
TG 8:0_18:1_18:2	0.556	1.618	1.689	0.611	2.865	0.895	0.755
TG 10:0_16:1_18:1	0.682	1.183	1.247	1.046	1.348	1.213	0.777
TG 10:0_16:1_18:1	0.651	1.902	1.385	0.976	1.314	1.321	0.795
TG 10:0_16:0_18:1	0.686	1.386	1.325	0.768	0.230	0.860	0.879
TG 10:0_14:0_20:0	1.041	1.258	1.549	0.950	2.270	0.757	0.326
TG 10:0_16:0_18:0	0.466	1.204	1.348	0.703	2.327	1.105	0.810
TG 10:0_17:1_18:1	0.967	1.776	0.937	0.711	0.779	1.250	0.935
TG 10:0_17:1_18:1	0.968	1.123	0.849	0.724	154.965	1.250	1.267
TG 10:0_17:0_18:2	0.839	1.398	1.689	0.756	0.196	1.300	1.090
TG 10:0_18:1_18:3	0.816	1.336	1.435	0.798	1.319	1.264	0.839
TG 10:0_18:1_18:3	0.841	1.663	1.480	0.823	0.561	1.307	0.865
TG 10:0_18:1_18:2	0.828	0.895	1.545	0.654	0.561	0.900	0.768
TG 12:0_16:0_18:3	0.829	1.209	1.544	0.775	0.424	0.839	0.997
TG 14:0_14:1_18:1	0.968	1.577	1.607	1.159	1.514	1.010	1.097
TG 14:0_14:1_18:1	0.818	1.494	1.461	0.948	1.701	1.395	0.995
TG 12:0_16:0_18:1	0.719	1.073	1.216	0.576	5.297	0.817	0.568
TG 12:0_16:0_18:1	0.702	1.191	1.289	0.716	5.274	1.134	0.688
TG 10:0_18:0_18:1	0.701	1.191	1.289	0.716	3.045	1.134	0.665
TG 14:0_16:0_16:0	2.548	6.194	2.994	0.885	1.291	2.941	2.790
TG 14:0_16:0_16:0	0.583	0.928	1.270	0.769	0.703	0.957	0.836
TG 14:0_16:0_17:0	0.585	0.738	0.923	0.300	1.249	1.027	0.861
TG 12:0_18:1_18:3	0.656	0.881	1.384	0.780	1.249	1.069	0.834

TG 16:0_14:1_18:2	0.742	1.103	1.539	0.737	1.314	1.325	0.865
TG 16:0_14:1_18:1	0.631	1.713	1.104	0.941	1.386	0.884	0.655
TG 14:0_16:0_18:1	0.983	1.267	1.663	1.068	0.268	1.604	0.965
TG 14:0_16:0_18:0	0.588	1.363	1.419	0.765	0.674	1.244	0.998
TG 14:0_18:1_18:3	0.449	1.109	0.989	0.729	1.526	1.125	0.816
TG 16:0_16:1_18:2	0.842	0.915	0.775	0.527	1.390	1.009	0.923
TG 16:1_16:1_18:1	0.807	1.263	1.048	0.690	1.417	1.075	0.810
TG 20:2_14:0_16:0	0.725	1.653	1.660	0.956	2.214	0.913	0.627
TG 16:0_16:1_18:1	0.631	1.420	1.417	0.816	2.656	0.945	0.818
TG 16:0_16:1_18:1	0.762	1.420	1.419	0.818	0.802	0.945	0.737
TG 16:0_16:0_18:1	0.894	1.218	1.562	0.939	1.134	1.107	1.031
TG 16:0_16:0_18:1	0.778	1.301	1.038	0.512	1.269	0.899	0.756
TG 14:0_18:0_18:1	1.014	1.591	0.993	0.880	2.248	0.986	0.532
TG 16:0_16:0_18:0	0.363	1.241	1.144	0.561	0.649	1.081	0.637
TG 16:0_17:1_18:1	0.787	1.582	1.855	0.915	1.465	1.850	0.719
TG 16:0_17:0_18:1	0.520	1.080	1.415	0.705	1.467	1.251	0.299
TG 16:0_18:2_18:3	0.985	1.254	1.526	0.605	1.469	1.032	0.922
TG 16:0_18:2_18:2	1.393	1.997	2.268	0.755	1.802	1.230	1.354
TG 16:0_18:1_18:2	1.078	1.424	1.601	0.639	1.219	1.257	0.903
TG 16:0_18:1_18:1	0.853	1.331	1.421	0.692	2.662	1.130	0.789
TG 16:0_18:0_18:1	0.631	1.090	1.355	0.648	1.779	1.301	0.768
TG 16:0_18:0_18:0	0.841	1.585	1.454	0.581	1.357	0.849	0.706
TG 17:0_18:1_18:1	0.807	1.404	1.427	0.714	1.468	1.037	0.807
TG 17:0_18:0_18:1	0.763	1.139	1.476	0.673	1.532	0.995	0.775
TG 18:2_18:2_18:3	0.922	1.329	1.174	0.532	0.030	1.182	0.957
TG 18:1_18:2_18:3	1.005	1.035	1.439	0.699	2.489	1.102	0.926
TG 18:1_18:2_18:3	1.052	0.835	1.978	0.694	1.447	1.531	0.970
TG 18:1_18:2_18:2	0.973	1.206	1.444	0.615	1.423	1.133	0.820
TG 18:1_18:1_18:2	0.854	1.174	1.468	0.611	8.320	1.146	0.851
TG 18:1_18:1_18:2	0.907	1.150	1.551	0.592	1.688	1.016	0.805
TG 18:0_18:1_18:2	0.890	1.316	1.522	0.718	2.863	0.775	0.803
TG 18:0_18:1_18:1	0.813	1.188	1.391	0.769	3.031	1.147	0.803
TG 18:0_18:0_18:1	0.641	1.019	1.008	0.630	1.689	0.766	0.560
TG 16:0_18:0_20:0	0.923	1.308	1.524	0.442	1.842	0.962	0.696
TG 18:1_18:1_19:1	0.941	1.315	1.638	0.697	1.254	1.100	0.804
TG 18:0_19:0_18:2	0.855	1.008	1.453	0.693	1.278	0.974	0.626
TG 18:0_19:0_18:1	0.613	1.359	0.964	0.710	1.467	1.115	0.390

TG 16:0_18:0_21:0	0.739	1.356	1.463	0.621	1.596	1.147	0.744
TG 16:0_18:1_22:5	0.873	1.326	1.603	0.718	1.663	1.300	0.821
TG 20:1_18:2_18:2	0.750	0.870	1.281	0.722	3.526	1.393	0.594
TG 18:0_18:1_20:3	0.910	0.738	1.588	0.562	4.730	1.202	0.882
TG 18:1_18:1_20:1	1.017	1.497	1.503	0.793	1.787	0.716	0.937
TG 18:0_18:1_20:1	1.197	2.033	1.847	0.845	1.603	1.574	0.809
TG 16:0_22:0_18:1	0.913	1.603	2.186	0.824	1.603	1.796	1.028
TG 16:0_18:0_22:0	0.722	1.341	1.207	0.590	1.580	0.515	0.732
TG 16:0_18:1_23:1	0.762	1.297	1.453	0.601	0.213	1.181	0.686
TG 16:0_18:0_23:1	0.743	1.398	1.455	0.658	1.545	1.114	0.825
TG 16:0_17:0_24:0	0.907	1.245	1.548	0.618	1.721	1.095	0.645
TG 22:1_18:2_18:2	0.972	1.231	1.514	0.602	1.818	1.065	0.834
TG 16:0_24:1_18:2	0.790	1.318	1.586	0.697	1.403	1.033	0.768
TG 16:0_24:0_18:2	1.112	1.482	1.369	1.014	1.581	0.891	0.897
TG 16:0_24:0_18:1	1.004	1.181	1.359	0.574	0.964	1.019	0.830
TG 16:0_18:0_24:0	0.833	1.249	1.303	0.660	1.635	1.000	0.813
TG 24:0_18:1_18:1	0.862	1.217	1.477	0.669	1.907	1.107	0.838
TG 16:0_26:0_18:1	0.787	1.065	1.402	0.712	2.374	1.137	0.570

Table S9: Substrate specificity factors depending on the lipase (A, E, G, J, K, M or O) in brioche (given as mean, n = 3).

	A	E	G	J	K	M	O
BisMePA 20:1_27:5	0.911	1.049	0.906	0.921	1.076	1.062	1.082
DG 30:6; O	1.205	1.296	1.049	1.319	0.716	1.362	1.316
DG 42:6; O	0.000	0.852	0.000	0.000	1.141	0.529	1.713
DG 6:0_12:1	0.697	0.662	0.896	0.823	1.188	1.066	0.662
DGDG 16:0_18:2	1.335	1.321	1.193	1.248	1.542	1.527	1.489
DGDG 18:2_18:3	1.226	1.342	1.040	0.957	1.280	1.070	1.381
DGDG 18:2_18:2	1.176	1.249	1.184	0.879	1.126	1.140	1.473
DGMG 18:2	0.449	0.829	1.063	0.922	0.740	0.596	0.593
LPC 16:1	0.927	1.145	1.063	1.079	1.176	0.966	1.138
LPC 18:3	1.290	1.446	1.313	1.354	1.415	1.232	1.190
LPC 18:1	0.761	1.031	1.038	0.964	0.559	0.992	0.814
LPC 18:0	0.696	0.933	0.866	0.685	0.672	0.875	0.777
LPC 22:6	0.000	1.612	1.425	1.195	1.003	0.702	--
LPE 16:0	1.096	1.210	1.109	0.983	1.190	1.137	1.369
LPE 18:2	0.990	1.269	1.093	1.061	1.174	1.149	1.326
MG 18:3	0.899	0.628	1.260	1.264	0.421	1.328	1.144
MG 18:2	1.049	0.931	1.122	1.002	1.031	0.737	0.872
MGDG 18:3_18:2	1.312	1.147	0.841	0.785	1.168	1.141	1.397
MGDG 18:2_18:2	1.381	1.147	0.979	0.882	1.184	1.126	1.589
MGMG 18:2	0.601	1.029	1.267	1.330	0.974	0.819	0.815
PC 26:4_22:6; O	1.026	0.985	0.932	1.109	1.334	1.069	1.050
PC 30:0	1.358	1.170	0.975	1.036	1.043	0.954	1.554
PC 32:2	1.062	1.064	1.159	1.127	1.126	1.154	1.130
PC 32:1	1.404	1.292	1.075	1.086	1.138	1.182	1.634
PC 32:0	1.253	1.176	1.028	0.930	1.136	1.010	1.361
PC 34:3	0.836	1.029	0.999	0.835	1.032	0.771	0.979
PC 34:2	0.997	1.115	1.049	1.025	1.040	1.048	1.194
PC 16:0_18:2	1.086	1.119	1.107	1.152	1.103	1.127	1.150
PC 16:0_18:1	1.876	1.183	1.150	1.070	1.408	1.226	1.909
PC 36:4	0.850	0.942	0.753	1.173	0.989	1.075	0.686
PC 18:2_18:2	0.956	1.021	1.005	0.999	1.019	1.033	0.999
PC 36:4	0.986	1.477	1.249	1.458	2.421	1.160	1.302
PC 18:1_18:2	0.772	0.823	0.910	0.728	0.764	0.856	0.562
PC 18:0_18:2	1.324	1.303	0.906	1.004	1.052	1.161	1.453

PC 36:2	1.234	1.266	1.041	0.925	1.097	1.083	1.385
PC 36:1	1.553	1.254	1.079	0.982	1.293	1.205	1.774
PC 38:6	1.437	1.459	1.247	1.151	1.223	1.120	1.510
PC 38:4	1.120	1.654	1.242	1.106	1.523	1.318	1.683
PE 18:2_18:2	0.938	1.459	1.237	1.179	1.339	1.184	1.353
PE 18:2_18:2	0.822	1.535	1.256	1.078	1.162	0.978	1.086
PEt O-26:3_18:4	1.077	1.188	1.316	1.073	1.277	1.085	1.166
PEt 16:1_16:1	1.222	1.219	1.020	0.950	1.204	1.314	1.085
PEt 10:0_22:1	0.947	0.781	1.047	0.697	0.876	0.892	0.726
PEt 24:6_18:2	1.628	1.519	0.807	1.195	1.432	1.314	2.054
PG O-21:0_20:4	0.741	1.043	0.826	0.831	0.494	1.055	0.349
PG O-28:5_20:3	1.072	1.144	1.842	1.449	1.359	1.864	0.953
PG O-28:3_20:4	1.051	1.099	1.169	1.030	1.012	1.016	0.907
PG 28:0_16:3	0.933	1.076	1.042	1.216	0.852	0.723	0.982
PG 28:1_22:5	1.180	1.393	0.959	1.401	1.082	1.435	1.351
PIP2 16:3_6:0	0.915	0.803	1.270	0.868	0.904	1.222	0.861
PIP3 P-6:0_16:3	0.901	1.074	1.330	1.216	1.352	1.209	1.068
SM d34:1	0.786	1.207	0.786	0.839	1.200	1.005	1.235
SM d40:1	0.316	0.483	1.255	1.090	0.000	1.636	1.642
SPB d18:0	1.380	0.829	0.982	0.933	0.722	1.069	0.959
SPB d14:1	3.242	0.773	1.378	1.058	0.792	1.654	1.275
SPB d16:1	2.539	1.060	1.197	1.636	0.878	1.390	0.964
SPB d20:1	0.971	1.648	1.515	1.344	1.147	1.789	1.435
TG 32:8_24:6; P	0.923	0.903	0.654	0.871	0.495	0.649	0.429
TG 25:8_36:9; P	1.165	1.396	1.015	1.245	1.208	1.469	1.450
TG 62:18; P	1.142	1.148	1.271	1.023	1.195	0.954	0.922
TG 38:10_24:7; P	1.173	1.160	0.874	1.224	0.592	1.038	0.881
TG 38:10_25:7; P	0.991	1.003	1.184	1.194	0.776	0.876	0.689
TG 22:7_23:7_23:7; P	0.852	1.190	1.134	1.121	1.210	0.959	1.108
TG 50:16_18:4; P	0.864	1.225	0.986	0.862	0.725	1.188	1.077
TG 43:13_26:7 ; P	0.906	1.021	0.985	0.893	1.096	1.062	0.937
TG 4:0_12:1_2:0; O	0.819	0.934	1.171	1.158	3.259	1.212	0.631
TG 28:8_24:7; O	1.030	0.993	0.950	1.040	1.362	1.244	0.972
TG 28:8_28:7; O	0.937	1.018	0.849	0.676	0.889	0.662	0.546
TG 47:15_22:6; O	1.045	1.102	1.056	0.828	1.195	1.138	1.001
TG 4:0_8:0_12:0	0.846	0.733	0.411	0.357	0.637	0.608	0.837
TG 4:0_4:0_18:1	1.122	1.085	1.071	0.721	1.060	1.100	1.230
TG 4:0_8:0_14:0	1.410	1.413	1.839	1.317	1.461	1.063	0.934

TG 4:0_6:0_18:1	1.496	1.464	1.533	1.525	1.290	0.765	1.765
TG 8:0_10:0_10:0	0.900	0.962	1.310	1.510	1.420	1.047	1.409
TG 4:0_10:0_14:0	1.014	0.923	0.874	0.829	0.667	0.758	1.001
TG 6:0_8:0_16:2	1.023	0.980	0.908	0.889	0.877	0.939	1.030
TG 4:0_8:0_18:1	1.033	0.984	1.165	0.780	0.880	0.749	1.287
TG 10:0_10:0_10:0	0.884	0.935	1.434	1.927	1.674	1.343	1.417
TG 4:0_12:0_14:0	1.258	1.146	1.180	1.196	0.652	1.024	1.004
TG 6:0_8:0_16:0	1.338	1.178	1.180	1.196	0.652	1.024	1.258
TG 4:0_10:0_18:2	1.031	1.059	0.730	0.947	0.900	1.022	1.090
TG 4:0_10:0_18:2	1.031	0.973	0.730	0.947	0.900	1.021	1.115
TG 4:0_10:0_18:1	0.980	0.957	0.861	0.770	0.824	0.852	0.961
TG 4:0_12:0_16:0	1.301	1.083	1.035	0.819	0.948	0.832	1.293
TG 4:0_12:0_16:0	0.894	0.984	1.055	1.010	1.187	1.081	1.115
TG 4:0_12:0_17:1	1.809	1.373	1.847	1.323	1.427	1.016	1.992
TG 4:0_12:0_18:3	1.201	0.979	1.170	1.175	1.169	1.134	0.997
TG 4:0_12:0_18:2	1.025	0.778	0.864	0.871	0.831	0.950	1.010
TG 6:0_10:0_18:1	0.943	1.098	1.060	0.995	0.841	0.710	1.138
TG 4:0_14:0_16:0	0.779	0.881	0.858	0.699	0.747	0.797	0.857
TG 4:0_14:0_17:1	1.292	0.429	0.448	0.526	0.709	0.797	1.481
TG 4:0_14:0_18:3	1.142	0.912	1.084	1.098	0.768	0.935	0.951
TG 4:0_14:0_18:2	0.885	0.795	1.059	0.529	0.687	0.816	0.907
TG 4:0_14:0_18:1	0.979	1.115	1.141	0.843	0.905	0.830	1.039
TG 6:0_14:0_16:0	0.983	1.041	1.609	2.472	2.513	1.515	1.884
TG 4:0_14:0_18:0	0.926	0.750	1.687	2.191	2.613	1.570	1.971
TG 4:0_16:0_16:0	1.072	1.174	1.105	1.087	2.382	0.976	2.840
TG 4:0_16:0_17:0	0.309	0.354	0.335	0.254	0.097	0.336	0.378
TG 4:0_16:0_18:3	0.827	0.979	0.947	0.766	0.868	0.899	0.992
TG 4:0_16:0_18:2	0.779	0.834	1.105	0.896	0.509	0.877	0.694
TG 4:0_16:0_18:1	0.463	0.545	0.497	0.642	1.003	0.866	0.510
TG 4:0_16:0_18:0	0.901	0.639	1.591	2.314	2.233	1.263	1.871
TG 6:0_16:0_16:0	1.030	1.398	1.023	1.200	1.044	0.974	0.939
TG 4:0_16:0_18:0	0.665	1.129	0.741	0.865	1.046	0.863	0.954
TG 8:0_14:0_16:0	0.929	1.282	0.817	1.271	1.422	0.894	0.734
TG 4:0_17:1_18:1	1.875	1.884	1.980	1.391	1.789	1.475	2.598
TG 4:0_17:0_18:1	1.927	0.818	1.159	0.710	11.920	1.373	0.655
TG 4:0_18:1_18:3	0.919	1.058	0.840	0.746	0.814	0.814	0.900
TG 4:0_18:1_18:2	0.909	0.992	1.061	0.852	0.900	1.012	0.941

TG 30:2_4:0_6:0	0.724	1.062	1.011	1.049	0.072	0.966	1.005
TG 4:0_18:1_18:1	0.865	1.045	1.339	1.155	1.217	1.723	1.059
TG 6:0_16:1_18:1	0.940	1.053	1.076	1.008	1.034	1.083	1.061
TG 6:0_16:0_18:1	0.895	0.934	1.186	0.905	1.224	0.910	1.034
TG 6:0_16:0_18:0	1.038	0.912	1.613	2.572	2.033	1.271	1.840
TG 6:0_16:0_18:0	1.394	1.075	1.556	1.483	1.393	1.009	1.216
TG 10:0_14:0_16:0	0.955	0.758	0.790	0.833	0.882	0.643	0.827
TG 6:0_17:0_18:1	0.958	0.981	0.973	1.012	0.940	0.970	1.049
TG 6:0_18:1_18:2	1.699	0.882	1.994	2.127	2.669	1.256	1.462
TG 18:2_12:0_12:0	0.916	1.262	1.070	2.206	1.373	0.957	1.298
TG 4:0_20:0_18:1	2.285	2.092	3.927	4.535	3.726	2.106	3.747
TG 8:0_16:0_18:1	1.035	0.357	0.791	0.271	0.021	0.999	0.310
TG 8:0_16:0_18:1	0.540	0.745	0.975	0.810	0.693	0.821	0.848
TG 10:0_12:0_20:0	0.939	0.871	0.959	2.014	1.781	1.066	1.583
TG 12:0_14:0_16:0	1.024	1.164	0.951	1.290	1.133	1.206	1.170
TG 8:0_18:0_17:1	0.255	0.267	0.351	1.186	0.335	0.800	0.445
TG 8:0_18:1_18:2	0.693	0.704	0.934	0.938	0.500	0.634	0.926
TG 28:2_6:0_10:0	0.158	1.108	0.847	0.249	1.226	1.057	1.016
TG 10:0_16:0_18:2	0.869	0.553	1.142	1.297	1.167	0.960	0.569
TG 10:0_16:1_18:1	0.953	0.934	0.897	0.991	1.024	0.851	0.962
TG 28:1_6:0_10:0	0.076	0.099	0.146	0.189	0.118	0.098	0.129
TG 12:0_14:0_18:1	1.620	1.120	1.353	1.018	1.598	1.119	1.048
TG 10:0_16:0_18:1	0.971	0.972	0.970	0.900	0.347	0.921	1.209
TG 10:0_16:0_18:0	0.779	1.103	1.032	0.941	0.898	0.845	0.600
TG 10:0_17:1_18:1	1.157	0.738	0.901	1.265	0.813	1.127	0.435
TG 10:0_18:1_18:3	0.995	1.019	0.986	1.008	0.956	0.982	1.018
TG 10:0_18:1_18:2	0.807	0.856	0.961	0.348	0.141	0.733	1.140
TG 10:0_18:1_18:1	1.074	0.865	0.829	1.177	1.010	0.743	0.853
TG 10:0_18:1_18:1	1.052	0.843	0.691	1.024	0.961	0.751	0.856
TG 10:0_18:0_18:1	0.936	0.780	1.005	1.053	0.958	0.913	1.262
TG 12:0_16:0_18:1	0.942	0.929	1.033	1.087	0.833	0.966	1.162
TG 14:0_16:0_16:0	1.078	1.302	2.172	1.311	0.944	1.039	3.387
TG 12:0_16:0_18:0	1.020	0.426	0.965	1.080	0.697	0.911	1.295
TG 12:0_17:1_18:1	1.150	1.052	1.191	1.187	0.759	1.013	1.010
TG 14:0_16:0_17:0	1.058	0.878	0.853	0.866	0.835	0.959	1.108
TG 12:0_18:1_18:3	0.965	0.999	1.008	1.007	0.976	1.019	1.072
TG 12:0_18:1_18:2	0.979	0.843	1.223	0.855	0.957	0.844	1.053
TG 16:0_14:1_18:1	2.962	3.288	2.436	3.376	2.666	3.209	2.147

TG 14:0_16:0_18:1	1.077	9.178	2.893	1.230	1.309	9.676	0.886
TG 14:0_16:0_18:0	0.949	1.346	0.935	0.936	0.253	0.769	1.511
TG 14:0_18:1_18:3	1.009	1.123	1.228	1.285	1.108	1.141	7.186
TG 14:0_18:1_18:2	0.829	1.022	0.869	0.931	0.970	0.808	0.215
TG 16:0_16:1_18:1	0.963	0.992	0.812	1.037	0.777	0.984	0.190
TG 16:0_16:0_18:1	1.248	0.330	1.096	0.143	1.142	0.658	1.121
TG 16:0_16:0_18:0	1.016	0.905	1.054	1.049	0.863	0.829	4.022
TG 16:0_17:1_18:1	1.406	1.124	1.131	1.157	1.133	0.805	0.804
TG 16:0_17:0_18:1	1.180	1.215	1.132	1.027	0.902	1.065	1.004
TG 16:0_18:2_18:3	0.944	1.121	1.008	1.039	0.999	1.034	2.406
TG 16:0_18:2_18:2	0.988	0.984	1.056	0.991	1.780	1.097	0.185
TG 16:0_18:1_18:2	0.880	1.033	0.954	0.969	1.026	0.966	0.242
TG 16:0_18:1_18:1	1.228	1.083	1.195	1.064	1.265	1.275	0.715
TG 16:0_18:1_18:1	1.773	1.354	1.567	1.698	1.090	1.785	1.761
TG 16:0_18:0_18:1	0.842	0.979	1.204	1.216	0.817	0.829	1.984
TG 16:0_18:0_18:0	0.776	0.951	0.717	0.730	0.672	0.586	9.787
TG 17:0_18:1_18:1	1.143	1.296	1.190	0.470	1.254	1.129	0.255
TG 17:0_18:0_18:1	0.742	1.171	1.130	1.076	1.189	1.159	1.201
TG 18:2_18:2_18:3	0.973	0.902	1.135	1.173	1.133	1.164	1.633
TG 18:2_18:2_18:3	0.999	1.299	1.172	1.392	1.128	1.162	1.315
TG 18:1_18:2_18:3	0.845	0.661	0.718	0.736	0.622	0.737	0.168
TG 18:1_18:2_18:3	0.907	0.885	0.874	0.846	1.206	0.741	4.321
TG 18:1_18:2_18:2	1.027	1.063	0.827	1.346	1.139	1.031	0.242
TG 18:1_18:1_18:2	0.801	1.054	0.979	0.989	1.090	1.084	0.692
TG 18:0_18:2_18:2	0.801	1.054	0.979	0.989	1.068	1.084	1.086
TG 18:1_18:1_18:2	0.731	1.076	1.165	1.141	1.127	1.136	1.360
TG 18:0_18:1_18:2	0.996	0.928	0.612	0.759	1.038	0.895	0.523
TG 18:1_17:2_20:4	1.296	1.051	0.883	0.511	15.901	1.532	43.377
TG 16:0_18:0_20:0	0.798	0.940	0.908	1.185	0.997	0.615	1.291
TG 18:1_18:1_19:1	0.946	1.221	1.209	1.157	1.165	1.014	0.381
TG 18:0_18:1_19:1	0.939	1.041	0.960	0.731	0.988	1.009	0.661
TG 16:0_21:0_18:1	1.008	1.159	1.027	1.147	0.774	1.039	1.233
TG 18:1_18:2_20:2	0.726	0.925	0.777	0.854	0.976	0.865	0.463
TG 18:1_20:1_18:2	1.041	0.976	1.306	1.169	1.244	1.260	1.129
TG 18:1_18:1_20:1	1.601	1.354	1.779	1.158	1.502	1.580	1.584
TG 18:0_18:1_20:1	0.856	1.055	1.032	1.022	0.848	0.857	0.816
TG 16:0_22:0_18:1	0.296	1.242	1.189	1.259	1.054	1.247	1.699

TG 16:0_18:0_22:0	0.906	0.994	0.946	0.800	0.953	0.851	1.914
TG 16:0_18:1_23:1	0.728	0.952	1.043	1.062	0.995	1.010	0.472
TG 16:0_18:0_23:1	0.915	0.976	1.015	0.982	0.979	0.987	1.051
TG 16:0_17:0_24:0	0.935	1.100	1.052	0.991	0.976	1.084	2.260
TG 22:0_18:1_18:2	0.946	0.961	1.002	1.104	1.038	0.965	0.917
TG 16:0_24:0_18:2	0.899	0.847	1.195	0.622	1.095	1.147	0.726
TG 16:0_24:0_18:1	2.612	1.811	2.787	2.692	5.840	2.794	4.126
TG 16:0_18:0_24:0	0.912	1.010	0.857	0.859	0.702	0.835	0.953
